# Obesity Prolongs the Inflammatory Response in Mice After Severe Trauma and Attenuates the Splenic Response to the Inflammatory Reflex

**DOI:** 10.3389/fimmu.2021.745132

**Published:** 2021-11-15

**Authors:** Fabian Gärtner, Adrian Gihring, Aileen Roth, Joachim Bischof, Pengfei Xu, Leonard Elad, Martin Wabitsch, Timo Burster, Uwe Knippschild

**Affiliations:** ^1^ Department of General and Visceral Surgery, Surgery Center, Ulm University Medical Center, Ulm, Germany; ^2^ Division of Pediatric Endocrinology and Diabetes, Department of Pediatrics and Adolescent Medicine, Ulm University Medical Center, Ulm, Germany; ^3^ Department of Biology, School of Sciences and Humanities, Nazarbayev University, Nur-Sultan, Kazakhstan

**Keywords:** obesity, immune response, severe trauma, inflammatory reflex, monocyte compartment, mass cytometry (CyTOF)

## Abstract

Thoracic traumas with extra-thoracic injuries result in an immediate, complex host response. The immune response requires tight regulation and can be influenced by additional risk factors such as obesity, which is considered a state of chronic inflammation. Utilizing high-dimensional mass and regular flow cytometry, we define key signatures of obesity-related alterations of the immune system during the response to the trauma. In this context, we report a modification in important components of the splenic response to the inflammatory reflex in obese mice. Furthermore, during the response to trauma, obese mice exhibit a prolonged increase of neutrophils and an early accumulation of inflammation associated CCR2^+^CD62L^+^Ly6C^hi^ monocytes in the blood, contributing to a persistent inflammatory phase. Moreover, these mice exhibit differences in migration patterns of monocytes to the traumatized lung, resulting in decreased numbers of regenerative macrophages and an impaired M1/M2 switch in traumatized lungs. The findings presented in this study reveal an attenuation of the inflammatory reflex in obese mice, as well as a disturbance of the monocytic compartment contributing to a prolonged inflammation phase resulting in fewer phenotypically regenerative macrophages in the lung of obese mice.

## Introduction

Trauma ranks as the leading cause of death in the population aged under 40 years, and about 20%–25% of fatal traumas are caused by thoracic injuries (1). Thoracic traumas are the third common cause of trauma-related mortality, while extra-thoracic injuries further increase the fatality rate (2). Therapeutic management as well as life expectancy after combined traumatic injuries are highly influenced by distinctive risk factors, including but not limited to obesity. In particular, obesity is known to be a risk factor of several diseases and is one of the most avoidable causes in preventable deaths (3). In recent studies, it was shown that obesity delays the regeneration processes of skeletal muscle and lung tissue due to an impaired regulation of extracellular matrix genes (4–10). In this context, we were able to show differences in cytokine and chemokine levels in obese mice in a preliminary analysis. These changes are most likely caused by compositional changes of the adipose tissue leading to altered production and secretion of anti- and pro-inflammatory adipocytokines, cytokines, and fatty acids. Obesity in combination with trauma increases the risk of comorbidities and mortality in patients and the impact on the immune response needs to be elucidated in this context (11, 12).

After a polytraumatic injury, the immune system responds in an intensive inflammatory manner that can, when not tightly regulated, result in severe complications, referred to as acute respiratory distress syndrome (ARDS), sepsis, or multiorgan failure (MOF). Due to damage-associated molecular patterns (DAMPs), such as DNA, ATP, and IL1α, neutrophils are rapidly recruited to the site of inflammation (13). During this process, DAMPs provoke the release of chemoattractants [CXC motif chemokine ligand 8 (CXCL8) and leukotriene B_4_ (LTB_4_)] by the surrounding tissue to induce chemotaxis of neutrophils (14, 15). Once they arrive at the site of injury, neutrophils phagocytose cellular debris and segregate further chemoattracting factors, for instance, azurocidin, attracting monocytes (16, 17).

Monocytes recruited to the site of trauma for immune defense and tissue remodeling subsequently differentiate towards macrophages to strengthen the pool of tissue-resident macrophages (18). In general, peripheral blood-derived monocytes are subcategorized into pro-inflammatory classical monocytes (Ly6C^hi^ in mice or CD14^+^CD16^-^ in humans) and the patrolling, anti-inflammatory non-classical monocytes (Ly6C^lo^ in mice or CD14^-^CD16^+^ in humans) (19–21). Human monocytes harbor a complementary subset of intermediate monocytes with the phenotype of CD14^+^CD16^+^, which represents a “transition” from the classical to the non-classical monocyte (22, 23). In humans and mice, the recruitment of pro-inflammatory monocytes is mainly driven by MCP-1 (CCL2) (24), mobilizing monocytes generated in the bone marrow (25) or from the spleen (26, 27). After a sterile injury, monocytes migrate to the tissue and turn not only into macrophages but also into dendritic cells (DCs), which promote inflammation and initiate tissue repair (28). Macrophage subsets are defined by their polarization to pro-inflammatory macrophages (M1), secreting cytokines to attract immune cells, and are responsible for clearing dead cells by phagocytosis, or anti-inflammatory macrophages (M2) (29). Whereby both subsets are important for tissue repair after traumatic injury (30, 31), a phenotype switch from M1 to M2 macrophages is pivotal for tissue regeneration (32). Both subtypes can be supported by infiltrating monocytes, which can either have phagocytic properties or promote the healing process (32). On the contrary, prolonged retention of pro-inflammatory subsets of monocytes and macrophages can lead to complications during the regeneration processes and prevent recovery (33).

During an immune response to sterile injury, it is important to address risk factors such as obesity, which influences the inflammatory response of the trauma, particularly by affecting distributions of immune cell subsets and their function. We hypothesize that obesity contributes to trauma-related inflammation resulting in a systemic inflammatory response and postulate that the prolonged inflammatory phase inhibits the replacement of inflammatory with regenerative macrophages. To this end, the significance of diet-induced obesity (DIO) to transform an immune-mediated inflammation to a regeneration process was addressed by using a previously established mouse model (4–6, 34, 35). C57BL/6J mice with or without DIO received a combined lung and muscle trauma. A combined trauma was selected to increase the clinical relevance of this model due to a simpler extrapolation of this study to the human where multiple traumas are often seen. In the mouse model, the circulating immune cells from these tissues were analyzed to define the association of inflammation with obesity by applying mass cytometry (cytometry by time-of-flight, CyTOF) as well as regular flow cytometry. Consistent with our hypothesis, a prolonged increase of neutrophils combined with an accumulation of pro-inflammatory monocytes was observed in obese mice causing a systemically extended inflammatory phase. This disturbance of the monocytic compartment results in an impaired switch from M1 to M2 macrophages in the traumatized lung. Furthermore, we report an impairment of the inflammatory reflex correlated with obesity by alterations in the vagus nerve-based cholinergic anti-inflammatory pathway of the spleen. We provide novel insights regarding the effects of DIO on the inflammatory reflex as well as the implication of DIO on the immune response after induced trauma *in vivo*, which is characterized by a systemic inflammatory response.

## Materials and Methods

### Animal Model and Breeding

Mouse samples were collected as part of animal studies, which were approved by the local and state authorities (Regierungspräsidium Tübingen, Ulm University/license numbers: 1183 and 1493). All animal experiments were carried out in accordance with local regulations and ARRIVE guidelines. A power analysis (nominal power: 0.8, nominal alpha: 0.025) to determine and calculate the sample size was conducted as part of the application of the animal experiments. The mouse model utilized female, non-genetically modified C57BL/6J mice to investigate the effects of DIO on the immune system after combined lung and muscle trauma induction. Therefore, parental animals received either a low-fat diet (LFD, 10% kcal fat; DI12450, Research Diets Inc., by their European distributor Brogaarden^®^ in Gentofte, Denmark) or a high-fat diet (HFD, 60% kcal fat, DI12492, Research Diets Inc., by their European distributor Brogaarden^®^ in Gentofte, Denmark) 1 week prior to breeding, resulting in a higher susceptibility of the litter to DIO and additionally influencing prenatal development (36). After 3 weeks, litters were weaned and received the parental diet (either LFD or HFD). Rearing conditions include a 12-h light/dark cycle at 22.5 ± 1°C with access to water and food *ad libitum*. The success of the DIO was assessed by weekly determination of the body weight. Final difference in body weight can be found in the supplement (**Supplementary Figure 5**). The induction of the combined trauma was carried out in 16 ± 1-week-old lean and obese female mice that were randomly grouped into control and trauma animals.

### Induction of a Combined Muscle and Lung Trauma

Animals were anesthetized utilizing a mixture of 2.5 vol% sevoflurane and 97.5 vol% oxygen in an anesthesia tube. During continued anesthesia using a rodent anesthesia mask, Buprenorphine (0.3 mg/ml) was injected subcutaneously. The chest area of the mice as well as the left upper hind leg were shaved. The experiment stopped at this time point for the control group. The trauma animals were used to simulate a combined trauma in the mouse model starting with the induction of a blunt skeletal muscle trauma followed by subsequent induction of a blunt thorax trauma. The left hind leg *extensor iliotibialis anticus* was used for muscle trauma induction with a drop tower apparatus described previously (4, 6, 37). The left hind leg was fixed between a scaffold and wedge and penetrated by the wedge after a weight (40 g) was dropped (height of 104 cm) on the leg leading to a blunt muscle injury. Limitation of penetration depth was achieved by using a spacer (3 mm) to prevent bone fractures. The thorax trauma was induced subsequently using a blast wave generator as described previously (4, 5, 34, 35). After the sternum was placed centrally under the cylinder, a high-speed valve connected to a gas cylinder containing compressed air with a pressure reducer (13 bar) was manually triggered. Blood and tissue samples of spleen, lung and muscle were collected from control and trauma mice 1 h, 6 h, 24 h, 72 h, and 192 h post trauma induction after the mice were sacrificed by carbon dioxide euthanasia.

### Cell Preparation for Cytometry Staining and ICS Assay

Blood was drawn directly from the heart and collected in two EDTA tubes; one tube was used for plasma extraction and one was used for cytometry analysis. After at least 30 min of resting, the EDTA-blood was centrifuged for 5 min at 1300 × *g* and the plasma was shock-frozen in liquid N_2_ and stored at −80°C. The second tube of EDTA-blood was incubated in RBC lysis buffer (Santa Cruz Biotechnology) for 10 min at room temperature (RT) and subsequently used for the specific staining. The spleen was harvested and dissolved by mechanical grinding (38) and subsequently filtered (100 µm, then 70 µm followed by 40 µm) to achieve a single-cell suspension. The suspension was incubated in RBC lysis buffer for 10 min at RT and subsequently used for the specific staining or the LPS stimulation.

The right lung (superior, middle, and inferior lobe) was harvested and enzymatically digested [45 min at 37°C in RPMI (Corning), 10% FBS (Life Technologies), 1% non-essential amino acids (Corning), 1% sodium-pyruvate (Corning), 1% L-glutamine (Corning), 1% penicillin-streptomycin (Life Technologies), and 1% HEPES buffer (Corning)]. This was followed by a filtration step to get to a single-cell suspension (100 µm, then 70 µm followed by 40 µm), which was incubated in RBC lysis buffer (Santa Cruz Biotechnology) for 10 min at RT and subsequently used for CyTOF staining.

The left hind leg *extensor iliotibialis anticus* was extracted. The muscle was first minced before incubated with HBSS containing 2 µg/ml collagenase A (Roche), 2.4 U/ml dispase I (Roche), 10 ng/ml DNase I (Roche), 0.4 mM CaCl_2,_ and 5 mM MgCl_2_ at 37°C for 90 min. This protocol has been shown to be efficient for subsequent mass cytometry staining by Spada et al. (39). After subsequent filtration steps (100 µm, 70 µm and finally 40 µm), cells were incubated for 10 min in RBC lysis buffer before being used for CyTOF staining.

### Flow Cytometry Staining

One milliliter of staining medium (1× PBS, 1% BSA, 2 mM EDTA, and 0.05% sodium azide) was added to the blood in the RBC lysis buffer. After centrifugation (300 × *g*, 7 min, RT) the cell pellet was resuspended in 1 ml of staining medium and counted. Cells (1.5 × 10^6^) were transferred and filled up to 1 ml with staining medium and centrifuged. Cells were resuspended in 100 µl of staining medium and stained on ice in the dark for 30 min. The master mix for blood and spleen samples analyzing immune subsets during the trauma response contained fluorescently labeled antibodies specific to CD11c (VioBlue, Miltenyi Biotec, 130-110-843), CD8a (VioGreen, Miltenyi Biotec, 130-109-330), CD3 (FITC, Miltenyi Biotec, 130-119-798), CD11b (PE, Miltenyi Biotec, 130-113-806), CD45R/B220 (PerCP-Vio700, Miltenyi Biotec, 130-102-218), CD4 (PE-Vio770, Miltenyi Biotec, 130-123-894), Ly-6C (APC-Vio770, Miltenyi Biotec, 130-111-919) and CD192/CCR2 (APC, Miltenyi Biotec, 130-119-658). The master mix staining for CHaT^+^ CD4 T cells contained labeled antibodies specific to ChAT (Alexa-Fluor 488, abcam, ab192465), CD3 (APC-Vio770, Miltenyi Biotec,130-119-793), CD11b (VioGreen, Miltenyi Biotec, 130-113-811), F480 (APC, Miltenyi Biotec, 130-116-525) and CD4 (PE, Miltenyi Biotec, 130-102-784). After washing twice with 1 ml of staining medium and subsequent centrifugation (300 × *g*, 7 min, RT), stained cells were resuspended in 500 µl of staining medium and immediately measured using a MACSQuant Analyzer 10 (Miltenyi Biotec) acquiring 500,000 events per measurement. FCS files were exported and analyzed using FlowJo v10.7.1 (BD).

### Mass Cytometry Staining

Tissue and blood were harvested and digested as described above. Single-cell suspensions were resuspended in CyFACS Buffer (1 × PBS (Rockland), 1% BSA, 2 mM EDTA, and 0.05% sodium azide) at a cell concentration of 3 × 10^7^ cells per ml. One hundred microliters of this dilution was used for staining and 100 µl of antibody mix was added to the cells (**Table 1**). All antibodies that were not purchased from Fluidigm were conjugated to the listed metal using the respective Maxpar^®^ X8 antibody labeling kit from Fluidigm following the instructions provided by the supplier.

**Table 1:** List containing all antibodies utilized for surface staining of mass cytometry samples.

Metal	Marker	Clone	Company	Item #	Dilution
**141Pr**	Ly-6G	1A8	Fluidigm	3141008	1:100
**143Nd**	CD69	H1.2F3	Fluidigm	3143004	1:100
**144Nd**	CD115	AFS98	Fluidigm	3144012	1:100
**145Nd**	CD4	RM4-5	Fluidigm	3145002	1:150
**146Nd**	F4/80	BM8	Fluidigm	3146008	1:150
**147Sm**	CD45	30-F11	Fluidigm	3147003	1:150
**148Nd**	CD11b[MAC1]	M1/70	Fluidigm	3148003	1:150
**149Sm**	CD19	6D5	Fluidigm	3149002	1:100
**151Eu**	CD25	3C7	Fluidigm	3151007	1:100
**152Sm**	CD3e	145-2C11	Fluidigm	3152004	1:100
**154Sm**	TER-119	TER119	Fluidigm	3154005	1:200
**155Gd**	CD206	MR5D3	FisherScientific	13246099	1:100
**PE**	Integrin a-7	3C12	Miltenyi Biotec	130-102-716	1:10
**159Tb**	TCRγδ	GL3	Fluidigm	3159012	1:100
**160Gd**	CD62L	MEL-14	Fluidigm	3160008	1:100
**161Dy**	CD90	His51	Miltenyi Biotec	130-094-524	1:100
**162Dy**	Ly-6C	HK1.4	Fluidigm	3162014	1:150
**164Dy**	CX3CR1	SA011F11	Fluidigm	3164023	1:150
**165Ho**	CD31	390	Fluidigm	3165013	1:150
**166Er**	CD117	2B8	Fluidigm	3166004	1:100
**167Er**	NKkp46	29A1.4	Fluidigm	3168003	1:100
**168Er**	CD8a	53-6.7	Fluidigm	3168003	1:150
**169Tm**	Ly6A/E	D7	Fluidigm	3169015	1:100
**170Er**	NK1.1	PK136	Fluidigm	3170002	1:150
**171Yb**	CCR2	475301	RnD	MAB55381	1:150
**172Yb**	CD86	GL1	Fluidigm	3172016	1:100
**174Yb**	CD127	A7R34	Fluidigm	3174013	1:100
**175Lu**	CD34	MEC 14.7	NovusBio	NB600-1071	1:100
**176Yb**	CD45R/B220	RA3-6B2	Fluidigm	3176002	1:100
**209Bi**	CD11c	N418	Fluidigm	3209005	1:100

Samples were gently mixed and incubated at RT in the dark for 30 min. Thereafter, samples were washed twice with 1 ml of CyFACS at 300 × *g* for 8 min. The cell pellet was resuspended in the leftover volume and the secondary antibody (anti-PE, Clone PE001, Fluidigm, 3156005, 1:100) was added as well as incubated at RT in the dark for 20 min. Cells suspended in 1 ml of CyFACS were then centrifuged at 300 × *g* for 8 min and subsequently fixed in 500 µl of PFA (3.7%) at RT for 30 min. Thereafter, 1 ml of ice-cold (-20°C) methanol was added to the samples and cells were stored at −80°C. Defrosted samples were washed twice with 1 ml of CyFACS (600 × *g* for 8 min) and subsequently stained with 50 µl of the following antibody mix for 20 min at RT in the dark (**Table 2**).

**Table 2:** Antibodies used for intracellular staining of mass cytometry samples.

Metal	Marker	Clone	Company	Item #	Dilution
**142Nd**	MyoD	SPM427	NovusBio	NBP2-32882	1:50
**150Nd**	pSTAT6	Tyr641	RnD	MAB55381	1:50
**153Eu**	pSTAT3	pSer727	SigmaAldrich	SAB4300034	1:50
**158Gd**	Foxp3	FJK-16s	Fluidigm	3165024	1:50
**163Dy**	pSTAT1	Y701	RnD	AF2894	1:50
**173Yb**	Pax7	HGH-16	Bosterbio	M00845-1	1:50

After the incubation period, samples were washed twice with 1 ml of CyPBS (600 × *g*, 8 min). The Cell-ID intercalator-Ir was diluted 1:2,000 in PBS, and 500 µl of this dilution was used for each sample. Samples were incubated at RT for 20 min. Cells were washed twice with CyPBS (600 × *g* for 8 min). After the last washing steps, cells were resuspended in 1 ml of freezing medium (RPMI, 10% DMSO) and stored at −80°C until day of acquisition. Samples were thawed on the day of acquisition and washed twice with 1 ml of CyFACS solution and three times with 1 ml of Millipore water (600 × *g* for 8 min). Samples were acquired at 300 events/s on a Helios (Fluidigm).

### Intracellular Cytokine Staining

Splenocytes were isolated from the spleen as previously described, and 2 × 10^6^ cells were seeded in 200 µl of complete RPMI medium supplemented with 10% FBS, 1% Pen-Strep, and 1% glutamine into wells of a deep-well 96-well plate. Each sample was split into an unstimulated control and six additional stimulation wells. The cells were rested overnight (14 h) at 37°C in a CO_2_ incubator. Thereafter, cells were stimulated with activation reagents as well as the secretion inhibitor (Brefeldin A, final concentration of 10 µg/ml). The samples were treated with one of the stimulation settings displayed in **Table 3**.

**Table 3:** Used conditions for different stimulation settings for ICS assay.

Stimulation settings	Additional information
**Unstim**	-
**PMA/Ionomycin**	100 ng PMA/2 µg Ionomycin
**PMA/Ionomycin**	100 ng PMA/2 µg Ionomycin
**[Low concentration of nicotine]**	10^-7^ mol/L nicotine
**PMA/Ionomycin**	100 ng PMA/2 µg Ionomycin
**[High concentration of nicotine]**	10^-6^ mol/L nicotine
**LPS**	10 µg/ml LPS
**LPS**	10 µg/ml LPS
**[Low concentration of nicotine]**	10^-7^ mol/L nicotine
**LPS**	10 µg/ml LPS
**[High concentration of nicotine]**	10^-6^ mol/L nicotine

The treated cells were incubated in a CO_2_ incubator at 37°C for 5 h. EDTA (final concentration of 2 mM) was added, and cells were incubated at RT for 15 min. Thereafter, cells were washed twice with PBS and centrifuged at 300 × *g* at RT for 8 min. Cells were stained with 1 µl of Zombie Green (423111, BioLegend) and incubated in the dark for 20 min followed by washing the cells once with PBS (centrifugation at 300 × *g* at RT for 8 min). Subsequently, cells were fixed in 200 µl of 3% PFA and incubated for 20 min in the dark at RT. After two washing steps with FACS buffer (centrifugation at 300 × *g*, 8 min, RT), surface markers were stained using CD3 (APC-Vio770, Miltenyi Biotec, 130-119-793), CD11b (VioGreen, Miltenyi Biotec, 130-113-811), and CD11c (VioBlue, Miltenyi Biotec, 130-110-843) and incubated at RT for 30 min in the dark. Samples were washed twice with 2 ml of FACS buffer, centrifuged for 300 × *g* at RT for 8 min, and resuspended in 500 µl of ice-cold permeabilizing solution (BioLegend Perm-2 Buffer, 421002). Thereafter, cells were centrifuged twice at 350 × *g* at 4°C for 8 min and the supernatant was discarded each time. Subsequently, each sample was stained using an anti-TNF-α antibody (FITC, Miltenyi Biotec, 130-124-212) and incubated on ice in the dark for 60 min. Thereafter, samples were washed three times with 2 ml of FACS buffer. Samples were resuspended in 200 µl of FACS buffer after the last washing step and immediately acquired using a MACSQuant Analyzer 10 (Miltenyi Biotec) acquiring 500,000 events per sample. FCS files were exported and analyzed using FlowJo v10.7.1 (BD).

### Mass Cytometry Data Acquisition and Processing

FCS files were generated and cleaned of the calibration beads (EQ™ Four Element Calibration Beads, Fluidigm #201078). Cells were identified with a gate on DNA double-positive events (^191^Ir and ^193^Ir) followed by a gate based on the residual and event length to a gate on single cells based on Gaussian gating (40). Immune cells were defined as CD45 positive. A gating example for this FCS file clean-up can be found in the supplement (**Supplementary Figure S6**).

### Data Analysis

FCS files generated during experiments using regular flow cytometry were analyzed using FlowJo 10.7.1. The gating scheme for the blood and spleen (**Supplementary Figure S7**), staining for ChAT^+^ CD4 T cells in the spleen (**Supplementary Figure S8A**), and the ICS staining (**Supplementary Figure S8B**) can be found in the supplement.

Mass cytometry data were analyzed using manual gating with FlowJo 10.7.1 as well as automated clustering approaches using R. The percentages achieved by manual gating were plotted against the populations identified by automated clustering using FlowSOM to determine differences in the identification of immune subsets. The graph can be found in the supplement (**Supplementary Figure S9**). The gating scheme for blood (**Supplementary Figure S10**), spleen (**Supplementary Figure S11**), lung (**Supplementary Figure S12**), and muscle (**Supplementary Figure S13**) can be seen in the supplement. In general, during manual gating and during the definition of the FlowSOM clusters, populations were identified as depicted in **Table 4**.

**Table 4:** General gating strategy for identified immune cell subsets and the used marker combination for definition.

Population name	Marker combination
**Neutrophils**	LIN-, Ly6G+, CD11b+
**B cells**	LIN-, CD19+
**T cells**	LIN-, CD3+
**γδT cells**	LIN^-^, CD3^+^, TCRγδ^+^
**CD4^+^ T cells**	LIN^-^, CD3^+^, CD4^+^, CD8^-^
**CD8^+^ T cells**	LIN^-^, CD3^+^, CD4^-^, CD8^+^
**NK cells**	LIN^-^, NK1.1^+^, NKkp46^+^
**NKT cells**	LIN^-^, NK1.1^+^, NKkp46^-^
**pDCs**	LIN^-^, CD11b^-^, CD11c^+^ Ly6C^+^
**Monocytes**	LIN^-^, CD11b^+^, CD115^+^, F480^-^
**Ly6C^hi^ Monocytes**	LIN^-^, CD11b^+^, CD115^+^, F480^-^, Ly6C^hi^
**Ly6C^lo^ Monocytes**	LIN^-^, CD11b^+^, CD115^+^, F480^-^, Ly6C^lo^
**Alveolar macrophages**	LIN^-^, F480^+^, CD11b^med^, CD11c^+^
**Interstitial macrophages (IM)**	LIN^-^, F480^+^, CD11b^+^, CD11c^-^
**CD11b^hi^ IM**	LIN^-^, F480^+^, CD11b^hi^, CD11c^-^, CD206^-^
**CD206^+^ IM**	LIN^-^, F480^+^, CD11b^+^, CD11c^-^, CD206+

Clustering approaches using R were carried out following the workflow and the code that was published by Nowicka et al. (41) and changed accordingly to fit the purposes of this study.

### LEGENDplex

The bead-based immunoplex assay from BioLegend, LEGENDplex, was performed to determine the level of several cyto- and chemokines in mouse plasma. The mouse inflammation panel (740446, BioLegend) was used with a V-bottom plate. Staining and acquisition were carried out according to the manufacturer’s specifications (LEGENDplex™, Mouse Inflammation Panel with V-bottom Plate, 10/2020 and Human Inflammation Panel with V-bottom Plate, 02/2021). The analysis was performed with the LegendPlex analysis software v8.0 (BioLegend).

### Statistical Information

GraphPad Prism 7.04 was used for statistical evaluation of the graphs. The tests used for each analysis are depicted below each graph. In general, a two-way ANOVA followed by an uncorrected Fisher’s LSD test (α = 0.05) was used if several time-dependent comparisons were performed. Whenever two time points were compared to each other, an unpaired Student’s *t*-test was used. Data are depicted as mean ± SEM and, if stated, baseline-corrected by calculation of the ratio. The following indicators were used for all statistical tests:. indicates *p* < 0.1, * indicates *p* < 0.05, ** indicates *p* < 0.01, *** indicates *p* < 0.001, and **** indicates *p* < 0.0001. Heatmaps either present normalized data using control mice as a reference for ratio calculation or depicting *z*-score normalized data to better emphasize the relative differences between the represented groups. The specific method is stated in the figure legend. For each graph, the sample sizes are depicted in the figure legend.

## Results

### DIO Effects Distribution of Adaptive and Innate Immune Populations

**Figure 1:**
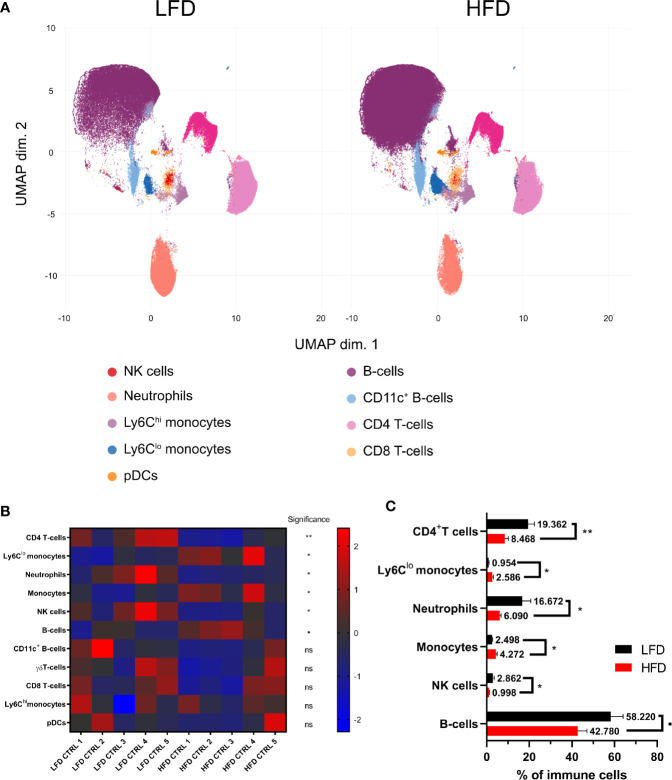
Influence of diet-induced obesity on circulating murine immune cells. Uniform manifold approximation and projection (UMAP) with clusters from FlowSOM analysis (Ly6G, CD115, CD4, CD11b, CD19, CD3e, TCRgd, Ly6C, NKp46, CD8a, NK1.1, B220, and CD11c) in mice receiving either low-fat diet (LFD) or high-fat diet (HFD) **(A)**. *Z*-score normalized heatmap indicating percentages of immune cell populations in lean and obese mice **(B)** as well as their actual percentages **(C)**. Statistics: unpaired two-tailed Student’s *t*-test to compare the populations between lean and obese mice. ▪ indicates *p* ≤ 0.1, * indicates *p* ≤ 0.05, ** indicates *p* ≤ 0.01. Sample sizes: LFD CTRL = 5, HFD CTRL = 5. Data are displayed as mean ± SEM. ns, not significant.

To address the hypothesis that DIO has an impact on the immune system, we conducted a phenotyping approach to define immune subpopulations in the peripheral blood of lean and obese mice by using manual and unsupervised machine-based gating strategies of CyTOF data. A principal component analysis (PCA)-based non-redundancy score (NRS) was performed to determine markers that contribute to the variability of the data set (42) (**Supplementary Figure S1A**). These calculations represent first indication in relation to variances in cell surface marker expression of immune cells between lean and obese mice with Ly6C, CD19, CD11b, Ly6G, and CD4 as the highest scoring markers based on NRS. Furthermore, CD115 demonstrated a clear separation in average expression between samples from lean and obese mice. The calculated differences based on NRS of the expression of lineage markers between samples from lean and obese mice were confirmed after clustering the data with FlowSOM (**Supplementary Figure S1B**) and was visualized *via* uniform manifold approximation and projection for dimension reduction (UMAP) as well as by analyzing the data set manually (**Figure 1A**). Besides a decrease of CD4^+^ T cells, neutrophils, and NK cells, an increase of total monocytes as Ly6C^lo^ anti-inflammatory monocytes was detectable among peripheral blood cells in obese mice in contrast to lean mice (**Figures 1B, C**).

### Peripheral Blood-Derived Myeloid Cells Differentiate the Immune Response to Trauma of Obese and Lean Mice

**Figure 2:**
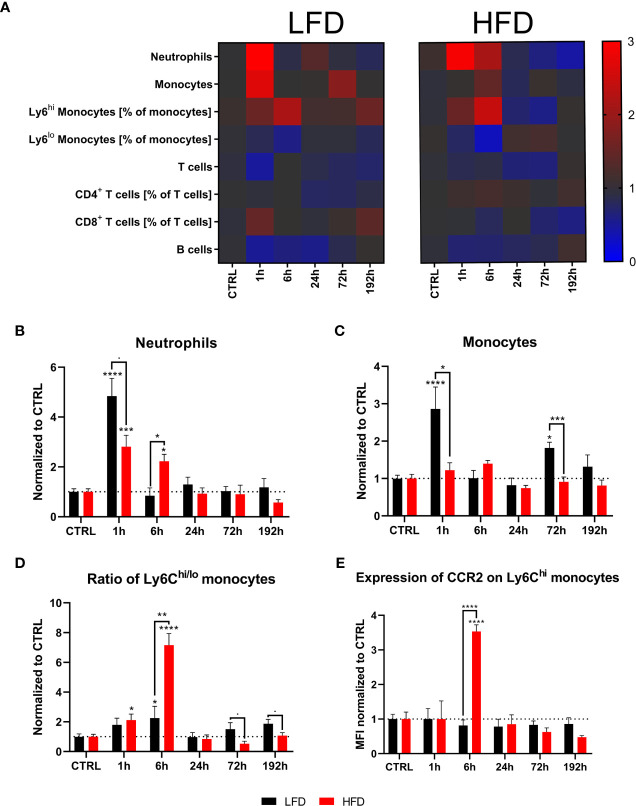
Immune subsets in the blood at defined time points during the first 192 h analyzed with flow cytometry. Baseline-corrected heatmap displaying the ratios of immune subsets to control level **(A)**. Baseline-corrected (ratio) timelapse for neutrophils **(B)**, monocytes **(C)**, the ratio of Ly6C^hi^ to Ly6C^lo^ monocytes **(D)**, and the expression of CCR2 on Ly6C^hi^ monocytes depicted as mean fluorescence intensity (MFI) **(E)**. Statistics: two-way ANOVA with an uncorrected Fisher’s LSD test as follow-up was used to compare the level to the respective CTRL, significance indicators are displayed directly above the bar; unpaired two-tailed Student’s *t*-test was used for comparison of lean and obese mice at a specific time point, significance indicators are displayed above a connector line. ▪ indicates *p* ≤ 0.1, * indicates *p* ≤ 0.05, ** indicates *p* ≤ 0.01, *** indicates *p* ≤ 0.001, **** indicates *p* ≤ 0.0001. Sample sizes: LFD CTRL = 8, LFD 1 h = 7, LFD 6 h = 5, LFD 24 h = 5, LFD 72 h = 5, LFD 192 h = 8, HFD CTRL = 7, HFD 1 h = 5, HFD 6 h = 5, HFD 24 h = 6, HFD 72 h = 5, HFD 192 h = 5. Data are displayed as mean ± SEM.

Next, the immune profile of lean and obese mice after a combined muscle and thorax trauma was analyzed at different time points using regular flow cytometry. A heatmap displays the means normalized to the respective control animals of several immune cell subsets from peripheral blood showing strong differences between lean and obese mice after trauma, specifically among the two myeloid populations of neutrophils and monocytes (**Figure 2A**). An early accumulation of neutrophils in lean as well as obese mice is detectable at 1 h after trauma induction (**Figure 2B**). While neutrophils of lean mice manage to return to steady state 6 h post trauma, obese mice exhibited a significantly increased number of neutrophils in the peripheral blood. This shift in neutrophil accumulation indicates a prolonged but not necessarily a stronger immune response of obese mice after trauma induction. In addition, monocytes were detected in these samples during an early response to the trauma; however, increased amounts of monocytes were only present in lean mice 1 h post trauma (**Figure 2C**). Notably, a second increase of monocyte numbers was detected in lean mice after 72 h and 192 h, respectively. Strikingly, levels of monocytes from obese mice did not change after trauma but were generally higher compared to lean mice (**Supplementary Figure S2**). Circulating monocytes were divided into pro-inflammatory Ly6C^hi^ monocytes and anti-inflammatory, patrolling Ly6C^lo^ monocytes for further characterization. Thereby, an enrichment of pro-inflammatory Ly6C^hi^ monocytes from obese mice 6 h post trauma was revealed (**Figure 2D**). As a result, a drastic change in the ratio of pro- to anti-inflammatory monocytes (Ly6C^hi^ to Ly6C^lo^ monocytes) (**Figure 2D**) and additionally an increase of cell surface CCR2 expression were observed in obese mice 6 h post trauma (**Figure 2E**). Thus, the phenotype of pro-inflammatory monocytes indicates a potential higher migration capacity following a CCL2 gradient.

### The Spleen Indicates Accumulation of Pro-Inflammatory Monocytes After Traumatic Injury

**Figure 3:**
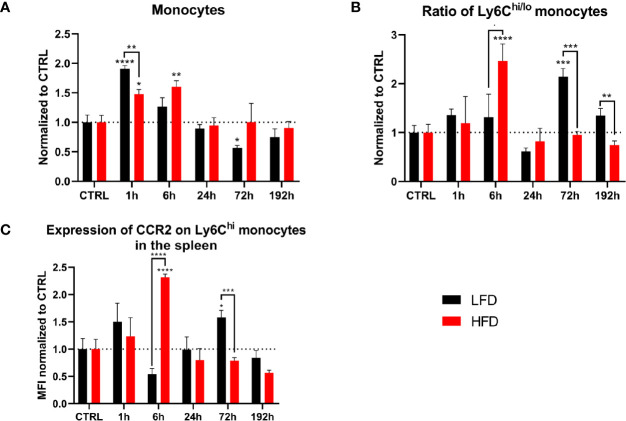
Myeloid cells in the spleen during the first 192 h analyzed by flow cytometry. Baseline-corrected (ratio) timelapse for monocytes **(A)**, the ratio of Ly6C^hi/lo^ monocytes **(B)**, and the expression of CCR2 on Ly6C^hi^ monocytes depicted as mean fluorescence intensity (MFI) **(C)**. Statistics: two-way ANOVA with an uncorrected Fisher’s LSD test as follow-up was used to compare the level to the respective CTRL, significance indicators are displayed directly above the bar; unpaired two-tailed Student’s *t*-test was used for comparison of lean and obese mice at a specific time point, significance indicators are displayed above a connector line. ▪ indicates *p* ≤ 0.1, * indicates *p* ≤ 0.05, ** indicates *p* ≤ 0.01, *** indicates *p* ≤ 0.001, **** indicates *p* ≤ 0.0001. Sample sizes: LFD CTRL = 13, LFD 1 h = 6, LFD 6 h = 5, LFD 24 h = 5, LFD 72 h = 6, LFD 192 h = 6, HFD CTRL = 14, HFD 1 h = 5, HFD 6 h = 5, HFD 24 h = 7, HFD 72 h = 5, HFD 192 h = 8. Data are displayed as mean ± SEM.

To identify whether the spleen acts as a reservoir for monocytes migrating to the traumatic tissue, we analyzed splenocytes of lean and obese mice after a traumatic injury. Conventional flow cytometry revealed an accumulation of monocytes in the spleen 1 h post trauma; however, the level of monocytes in the spleen was significantly higher in lean compared to obese mice (**Figure 3A**). Subsequently, the numbers of monocytes in the spleen of lean mice decreased to baseline levels after 6 h, in contrast to obese mice requiring more time (24 h) to restore to steady state (**Figure 3A**). Moreover, obese mice exhibited a higher ratio of pro- to anti-inflammatory monocytes after 6 h (**Figure 3B**), which can be attributed to an increased amount of Ly6C^hi^ monocytes (**Supplementary Figure S3A**). Lean mice exhibited a higher Ly6C^hi/lo^ ratio 72 h post trauma, caused by a declined number of Ly6C^lo^ monocytes (**Supplementary Figure S3B**). These findings indicate migration of Ly6C^lo^ monocytes from the spleen into the periphery in lean mice. The second response of monocytes to the trauma was not detected in the blood or spleen of obese mice. Remarkably, the surface expression of CCR2 on Ly6C^hi^ monocytes was increased in obese mice during the early response 6 h post trauma and in lean mice during the secondary response 72 h post trauma (**Figure 3C**). These data indicate that recruitment of monocytes from the spleen to the site of inflammation is distorted in obese mice post trauma.

### Obesity-Related Key Signature of CCR2^+^CD62L^+^Ly6C^hi^ Monocytes in the Blood and Spleen of Obese Mice

**Figure 4:**
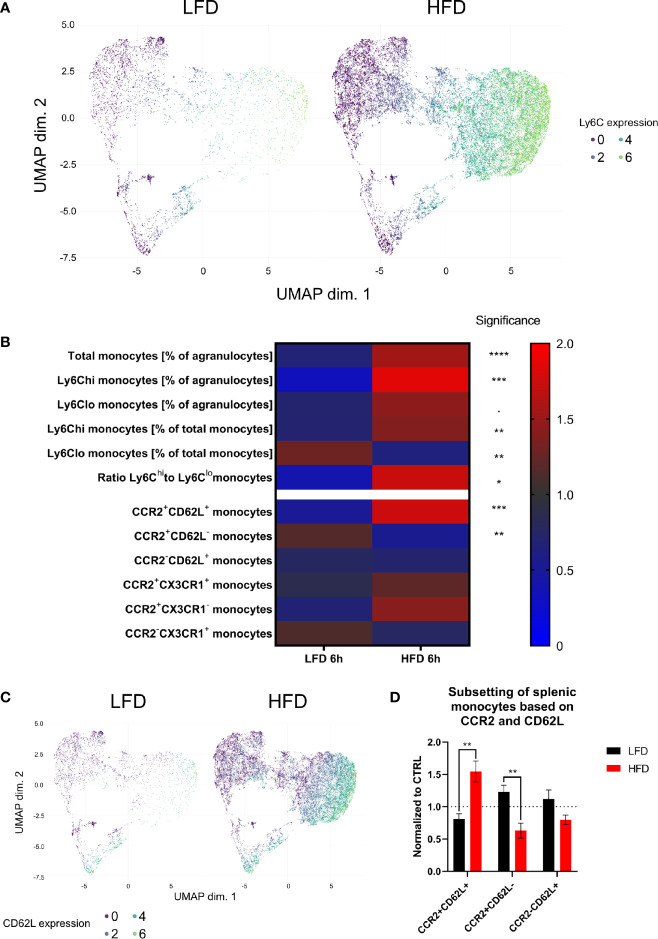
CyTOF analysis of circulating and splenic monocytes 6 h post trauma. Uniform manifold approximation and projection (UMAP) of monocytes (LIN^-^, CD115^+^, CD11b^+^) was generated based on the expression of CD115, CD11b, CD62L, Ly6C, CX3CR1, and CCR2 and colored by the expression of LY6C **(A)**. Heatmap showing various monocytes populations (normalized by ratio to mean) between mice receiving either low-fat diet (LFD) or high-fat diet (HFD) 6 h post trauma **(B)**. UMAP of monocytes (LIN-, CD115+, CD11b+) was generated based on the expression of CD115, CD11b, CD62L, CX3CR1, and CCR2 and colored by the expression of CD62L **(C)**. Comparison of splenic monocyte subsets (normalized to CTRL) based on the expression of CCR2 and CD62L between mice receiving either low-fat diet (LFD) or high-fat diet (HFD) 6 h post trauma **(D)**. Statistics: unpaired two-tailed Student’s *t*-test was used for comparison of lean and obese mice at a specific time point, significance indicators are displayed above a connector line. ▪ indicates *p* ≤ 0.1, * indicates *p* ≤ 0.05, ** indicates *p* ≤ 0.01, *** indicates *p* ≤ 0.001, **** indicates *p* ≤ 0.0001. Sample sizes: LFD 6 h = 6, HFD 6 h = 4. Data are displayed as mean ± SEM.

We used deep-profiling approaches utilizing mass cytometry to verify the above data by analyzing and comparing monocytes and neutrophils from the blood and spleen of lean and obese mice after traumatic injury. Six hours post trauma demonstrated the highest influence of DIO on the level of circulating myeloid cells, mainly Ly6C^hi^ monocytes and neutrophils, an observation that could be confirmed with our mass cytometry data (**Supplementary Figure S4**). Since monocytes are highly responsive to inflammatory milieus, they were analyzed at a higher resolution. Pre-gated monocytes (LIN^-^, CD115^+^, CD11b^+^) were clustered utilizing FlowSOM and visualized by UMAP by expression of CD11b, CD115, CD62L, CCR2, CX3CR1, and Ly6C to substantiate the results in more detail. As expected, unsupervised analysis of mass cytometry data and regular flow analysis confirmed the finding of an increased expansion of Ly6C^hi^ monocytes from the peripheral blood of obese mice 6 h post trauma (**Figure 4A**). Additionally, higher counts of CCR2^+^CD62L^+^ blood monocytes were detected in obese mice in contrast to lean mice by mass cytometry analysis (**Figure 4B**). These CCR2^+^CD62L^+^ monocytes can mainly be found among the pro-inflammatory Ly6C^hi^ monocytes in obese mice (**Figure 4C**). While obese mice exhibit increased levels of CCR2^+^CD62L^+^Ly6C^hi^ monocytes, lean mice harbor increased levels of CCR2^+^CD62L^-^Ly6C^hi^ monocytes 6 h post trauma. CD62L is important for migration of monocytes due to the binding of CD62L to the endothelium of blood vessels (43). Similarly, when monocytes from the spleen of obese mice 6 h post trauma were analyzed, higher levels of CCR2^+^CD62L^+^ monocytes were detected (**Figure 4D**). Collectively, mass cytometry strengthened the observations made with conventional flow cytometry and further indicates an increased quantity of inflammation associated CCR2^+^CD62L^+^Ly6C^hi^ monocytes during the immune response to the trauma in obese mice.

### Distinct Pro-Inflammatory Cytokine Patterns in Obese Mice 6 h Post Trauma

**Figure 5:**
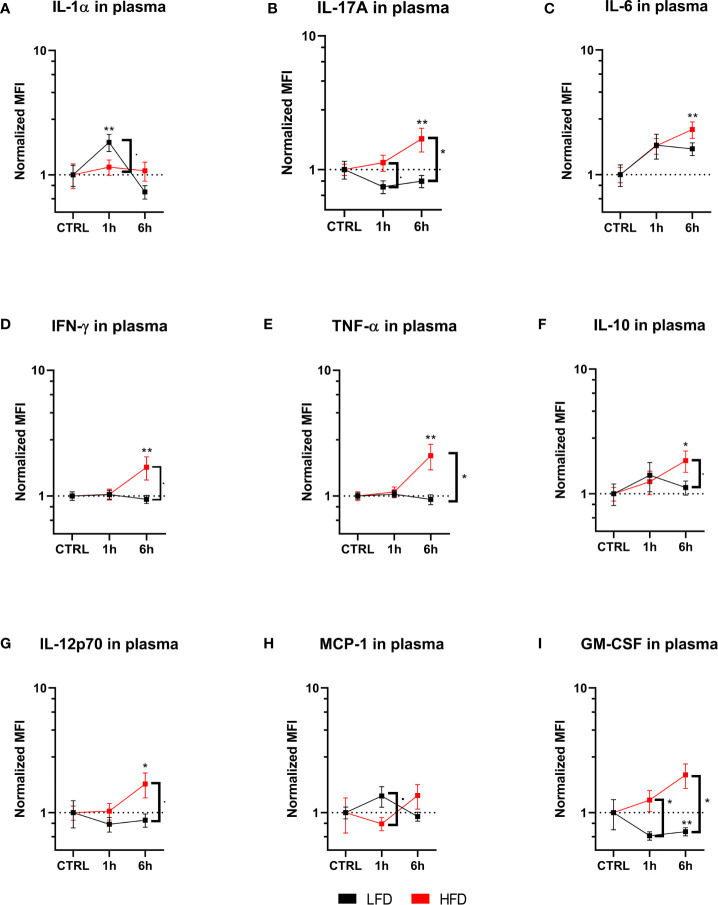
Immunoplex assays analyzing various pro-inflammatory molecules in plasma during the early response up to 6 h. Timelapse showing the level of IL-1α **(A)**, IL-17A **(B)**, IL-6 **(C)**, IFNγ **(D)**, TNF-a **(E)**, IL-10 **(F)**, IL-12p70 **(G)**, MCP-1 **(H)** and GM-CSF **(I)**. depicted as mean fluorescence intensity (MFI) and normalized to the respective control. Statistics: two-way ANOVA with an uncorrected Fisher’s LSD test as follow-up was used to compare the level to the respective CTRL, significance indicators are displayed directly above the bar; unpaired two-tailed Student’s *t*-test was used for comparison of lean and obese mice at a specific time point, significance indicators are displayed next to the connector line. ▪ indicates *p* ≤ 0.1, * indicates *p* ≤ 0.05, ** indicates *p* ≤ 0.01. Sample sizes: Each group at each time point: *n* = 5. Data are displayed as mean ± SEM.

The cytokine profile of lean and obese mice was analyzed post trauma to further categorize the immune response in peripheral blood. Lean mice initially showed an increase of IL-1α 1 h post trauma, which follows the observation of an increased level of neutrophils in the blood of these mice. In contrast, obese mice did not show any changes of IL-1α levels during the indicated time points (**Figure 5A**). On the other hand, IL-6, IL-17A, IFN-γ, and TNF-α were significantly increased in obese mice 6 h post trauma (**Figures 5B–E**). TNF-α can be produced by neutrophils and pro-inflammatory Ly6C^hi^ monocytes, which is an indication that besides showing a phenotypical pro-inflammatory profile, the produces number of circulating Ly6C^hi^ monocytes in obese mice produce pro-inflammatory cytokines, namely, TNF-α (44, 45). Even though differences between lean and obese mice did not elicit substantial changes in the cytokine profile, pro-inflammatory cytokines were increased in obese mice 6 h post trauma. Similarly, immunoregulatory cytokines IL-10 (**Figure 5F**) and IL-12p70 (**Figure 5G**) were increased in obese mice 6 h post trauma. However, the differences in IL-10 and IL-12p70 levels between lean and obese mice were not statistically significant. MCP-1, which is responsible for the recruitment of monocytes to the site of inflammation (24), was increased in lean mice 1 h post trauma but did not reach statistical significance (**Figure 5H**); similar results were also received for obese mice 6 h post trauma. Furthermore, the quantities of GM-CSF were increased in obese mice when compared to those of lean mice 1 h and 6 h post trauma (**Figure 5I**). This is in accordance with the higher ratio of pro- to anti-inflammatory monocytes in the peripheral blood of obese mice 6 h post trauma, since GM-CSF promotes a pro-inflammatory profile in monocytes of both humans (46) and mice (47).

Obese mice retain more circulating neutrophils and inflammation-associated CCR2^+^CD62L^+^Ly6C^hi^ monocytes, while showing an increased pro-inflammatory cytokine profile compared to lean mice. These factors contribute to an enhanced pro-inflammatory immune response and systemic inflammation after traumatic injury in obese mice.

### Prolonged and Increased Infiltration of Pro-Inflammatory Myeloid Populations in the Lung and the Muscle of Obese Animals During Early Time Points

**Figure 6:**
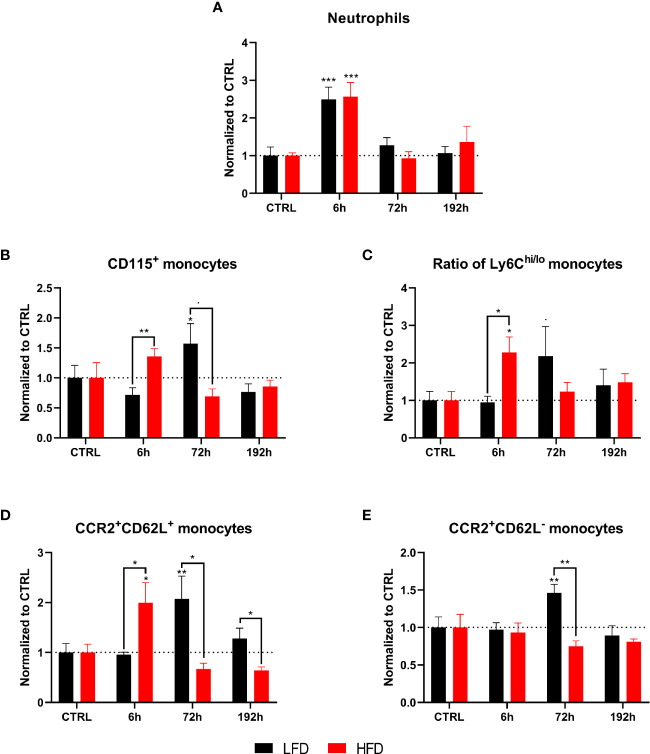
CyTOF analysis of immune population in the lung of lean and obese mice after trauma. Baseline-corrected (ratio) timelapse for neutrophils **(A)**, monocytes **(B)**, the ratio of Ly6C^hi/lo^ monocytes **(C)**, as well as CCR2^+^CD62L^+^ monocytes **(D)**, and CCR2^+^CD62L^-^ monocytes **(E)**. Statistics: two-way ANOVA with an uncorrected Fisher’s LSD test as follow-up was used to compare the level to the respective CTRL, significance indicators are displayed directly above the bar; unpaired two-tailed Student’s *t*-test was used for comparison of lean and obese mice at a specific time point, significance indicators are displayed next to the connector line. ▪ indicates *p* ≤ 0.1, * indicates *p* ≤ 0.05, ** indicates *p* ≤ 0.01, *** indicates *p* ≤ 0.001. Sample sizes: LFD CTRL = 5, LFD 6 h = 5, LFD 72 h = 6, LFD 192 h = 5, HFD CTRL = 5, HFD 6 h = 5, HFD 72 h = 4, HFD 192 h = 5. Data are displayed as mean ± SEM.

To determine the infiltration of immune cells after trauma, lung and muscle tissue of lean and obese mice was analyzed by mass cytometry. Neutrophils accumulated in the lung of mice 6 h post trauma and both groups returned to baseline level 72 h post injury. Both lean and obese mice showed the same infiltration pattern but no differences in neutrophil infiltration into the lung after trauma induction was detected between the two groups (**Figure 6A**). The observed increase of circulating neutrophils in the peripheral blood of obese mice did not influence the migration pattern of neutrophils into the lung. A high number of infiltrating monocytes into the lung of obese mice was detected 6 h post trauma (**Figure 6B**), which was accompanied by an increased ratio of Ly6C^hi^ to Ly6C^lo^ monocytes (**Figure 6C**) as well as increased levels of CCR2^+^CD62L^+^ monocytes (**Figure 6D**). These differences might be due to an upregulation of CD62L expression on CCR2^+^ blood monocytes or a reduced shedding of CD62L after activation resulting in an increased migratory potential to the site of inflammation. Notably, the population of infiltrating monocytes is characterized by CD62L expression, since an accumulation of CCR2^+^CD62L^-^ monocytes in the lung of obese mice was not observed 6 h post trauma (**Figure 6E**). On the contrary, lean mice showed an increase of the Ly6C^hi^/Ly6C^lo^ monocyte ratio that did not reach statistical significance (**Figure 6C**) 72 h post trauma. These infiltrating monocytes expressed the chemokine receptor CCR2. In contrast to obese mice—in which the infiltrating monocytes only displayed a simultaneous expression of CD62L and CCR2 6 h post trauma—the infiltrating monocytes of lean mice either retained a CCR2^+^CD62L^+^ or a CCR2^+^CD62L^-^ phenotype (**Figures 6D, E**).

**Figure 7:**
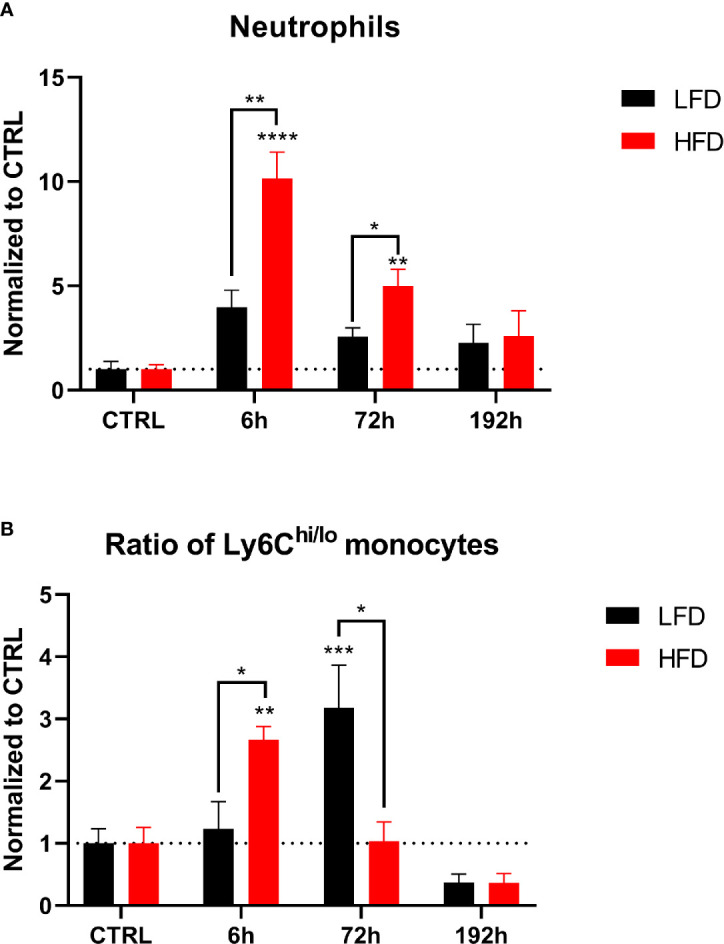
CyTOF analysis of immune population in the muscle of lean and obese mice after trauma. Baseline-corrected (ratio) timelapse for neutrophils **(A)** and the ratio of Ly6C^hi/lo^ monocytes **(B)**. Statistics: two-way ANOVA with an uncorrected Fisher’s LSD test as follow-up was used to compare the level to the respective CTRL, significance indicators are displayed directly above the bar; unpaired two-tailed Student’s *t*-test was used for comparison of lean and obese mice at a specific time point, significance indicators are displayed next to the connector line. * indicates *p* ≤ 0.05, ** indicates *p* ≤ 0.01, *** indicates *p* ≤ 0.001, p**** indicates *p* ≤ 0.0001. Sample sizes: LFD CTRL = 4, LFD 6 h = 6, LFD 72 h = 6, LFD 192 h = 4, HFD CTRL = 5, HFD 6 h = 4, HFD 72 h = 4, HFD 192 h = 4. Data are displayed as mean ± SEM.

Next, we determined the migration of neutrophils into muscle tissue. High numbers of neutrophils were detected in obese mice 6 h post trauma (**Figure 7A**). An early increase of the ratio of Ly6C^hi^ to Ly6C^lo^ monocytes was found in obese animals 6 h post trauma, inversely to lean mice, which indicated an increased ratio 72 h post trauma (**Figure 7B**). Taken together, infiltration of myeloid immune cells into the lung and the muscle show differences between lean and obese mice regarding the time point of infiltration.

### Lung Macrophage Populations Demonstrate an Impaired Switch From M1 to M2 Macrophages During the Trauma Response in Obese Mice

**Figure 8:**
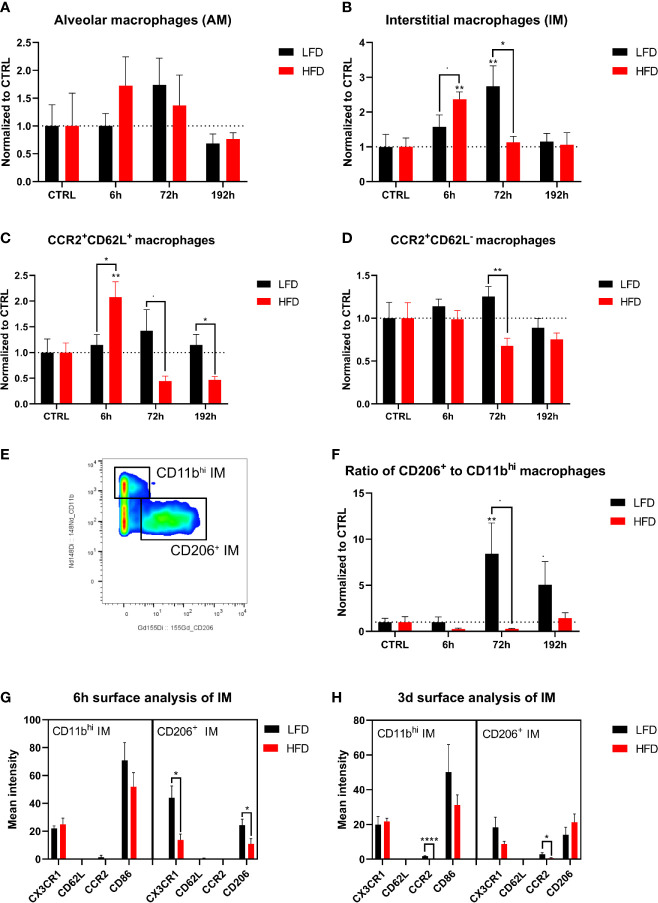
CyTOF analysis of macrophage population in the lung of lean and obese mice after trauma. Baseline-corrected (ratio) timelapse for alveolar macrophages (AM) **(A)**, interstitial macrophages (IM) **(B)**, CCR2^+^CD62L^+^ macrophages **(C)**, and CCR2^+^CD62L^-^ macrophages **(D)**. A gating example is showing the differentiation between CD11b^hi^ and CD206^+^ macrophages **(E)** and is used to calculate the ratio of CD206^+^ to CD11b^hi^ macrophages over the timelapse of the study **(F)**. Analysis of the surface markers CX3CR1, CD62L, CCR2 as well as CD86 or CD206 on CD11b^hi^ and CD206^+^ macrophages is depicted 6 h post trauma **(G)**, as well as 72 h post trauma **(H)**. Statistics: two-way ANOVA with an uncorrected Fisher’s LSD test as follow-up was used to compare the level to the respective CTRL, significance indicators are displayed directly above the bar; unpaired two-tailed Student’s *t*-test was used for comparison of lean and obese mice at a specific time point, significance indicators are displayed next to the connector line. indicates *p* ≤ 0.1, * indicates *p* ≤ 0.05, ** indicates *p* ≤ 0.01, p**** indicates *p* ≤ 0.0001. Sample sizes: LFD CTRL = 5, LFD 6 h = 5, LFD 72 h = 6, LFD 192 h = 5, HFD CTRL = 5, HFD 6 h = 5, HFD 72 h = 4, HFD 192 h = 5. Data are displayed as mean ± SEM.

Lung-derived macrophages were phenotyped and categorized by their cell surface expression profiles to determine the influence of DIO on the migration of monocytes from the blood to the lung after trauma. Macrophages were subdivided into alveolar macrophages (AM) and interstitial macrophages (IM) (48, 49). The number of AMs (LIN^-^, F480^+^, CD11b^lo^, CD11c^+^) did not change significantly after trauma (**Figure 8A**). Obese mice had an increased number of IMs (LIN^-^, F480^+^, CD11b^+/hi^, CD11c^+^) in the lung 6 h post trauma, while lean mice indicate higher numbers of IMs 72 h post trauma (**Figure 8B**). IMs were further characterized by the expression of CCR2, CD62L, CD206, and CD11b. An expansion of CCR2^+^CD62L^+^ IMs was detected in obese mice 6 h post trauma and subsequently dropped below baseline level at later time points, which leads to increased levels of this population in lean mice starting from 72 h post trauma (**Figure 8C**). Increased level of CCR2^+^62L^+^ IMs was accompanied by a simultaneous significant increase of CCR2^+^CD62L^-^ IMs in lean mice (**Figure 8D**). Lung IMs comprise the same expression patterns regarding CCR2 and CD62L during the immune response that was observed on the circulating blood monocytes in lean and obese mice indicating that these cells migrate from the periphery to the traumatized tissue and differentiate to monocyte-derived macrophages to enhance the pool of tissue-resident macrophages in the lung.

Further division of IMs to pro-inflammatory CD11b^hi^CD206^-^ (M1-like) IMs and anti-inflammatory CD11b^+^CD206^+^ (M2-like) IMs (50, 51) enabled the calculation of the ratio of phenotypically anti-inflammatory and pro-inflammatory IMs (**Figure 8E**). As a result, an increased anti-inflammatory response of lean mice was observed 72 h and 192 h post trauma based on an increased ratio of anti- to pro-inflammatory macrophages (**Figure 8F**). This increased ratio was absent in obese animals, in which an expansion of regenerative macrophages was not detectable either 72 h or 192 h post trauma, indicating an impaired ability of conversion from M1 to M2 phenotype in these mice. Additional analysis of the cell surface markers CX3CR1, CD62L, CCR2, and CD206 or CD86 6 h and 72 h post trauma revealed a higher expression of CX3CR1 as well as CD206 on anti-inflammatory macrophages in lean mice 6 h post trauma compared to obese mice (**Figure 8G**). Furthermore, lean mice showed increased expression of CCR2 on both CD11b^hi^ and CD206^+^ macrophages 72 h post trauma (**Figure 8H**). In summary, obese mice exhibited an impaired switch of M1 to M2 macrophages post trauma.

### Impaired Splenic Inflammatory Reflex in Obese Mice Contributes to Prolonged Systemic Inflammation

**Figure 9:**
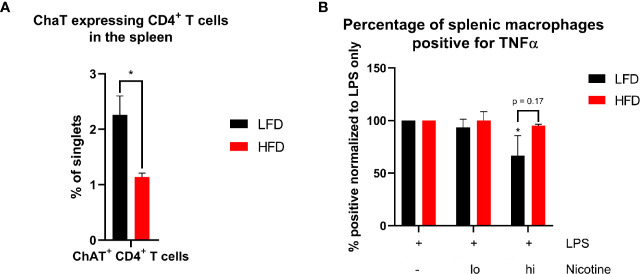
Analysis of essential components of signal transduction of the inflammatory reflex in the spleen. Occurrence of ChAT^+^ CD4^+^ T cells in the spleen of lean and obese mice **(A)**. Statistics: Comparison of lean and obese mice was achieved by an unpaired two-tailed Student’s *t*-test. Comparison of TNF-α^+^ splenic macrophages after LPS stimulation with or without different concentrations of nicotine treatment to the control **(B)** were analyzed using a two-way ANOVA with repeated measurements followed by an uncorrected Fisher’s LSD test. * indicates *p* ≤ 0.05. Sample sizes: Each group at for each analysis and condition: n = 5. Data are displayed as mean ± SEM.

Ly6C^lo^ monocytes showing different migration patterns in lean and obese mice as well as differences regarding TNF-α levels in the plasma of lean and obese mice implicate the involvement of the inflammatory reflex as a contributor to the described changes during the immune response. The inflammatory reflex describes a protective response to injury by limiting inflammation *via* stimulation through the vagus nerve (52) leading to a blockade of pro-inflammatory cytokine release (such as TNF-α) by the spleen, liver, and the gastrointestinal tract (53). A subset of CD4^+^ T cells that express the choline acetyltransferase (ChAT) is crucial for the neural circuit and the respective impulse of the vagus nerve (54). ChAT-expressing spleen-resident CD4^+^ T cells were significantly decreased in obese mice (**Figure 9A**), which suggests a decreased signal transduction in the spleen after signaling through the vagus nerve. The impulse of the vagus nerve induces the release of acetylcholine (ACh) from ChAT-expressing CD4^+^ T cells, which binds to the nicotinic acetylcholine receptor alpha 7 (α7nAChR) of macrophages and inhibits, for instance, the production of TNF-α (55). Since nicotine has the same anti-inflammatory effect as stimulation through the vagus nerve (55, 56), nicotine was used to simulate signaling through α7nAChR of macrophages. Splenocytes were stimulated with lipopolysaccharide (LPS) and treated with different nicotine concentrations to evaluate the TNF-α secretion of macrophages. In contrast to macrophages from obese mice, the segregation of TNF-α was decreased in lean mice by increasing amounts of nicotine (**Figure 9B**). Thus, obese mice comprise reduced numbers of spleen-derived ChAT-expressing CD4^+^ T cells and an impaired response to nicotine stimulation imitating the inflammatory reflex by the vagus nerve. By failing to convert signaling of the inflammatory reflex to a reduced production of pro-inflammatory cytokines, both described obesity-related impairments contribute to a distorted immune response in obese mice and to the prolonged systemic inflammatory phase after trauma.

## Discussion

In this study, a comprehensive approach to characterize immune cell subsets in the peripheral blood, spleen, and the traumatized tissues was conducted using sample material from lean and obese mice after a combined thorax and muscle trauma. Obese mice exhibited a prolonged systemic pro-inflammatory response to the traumatic injury, based on an increased number of circulating neutrophils and inflammation-associated CCR2^+^CD62L^+^Ly6C^hi^ monocytes in combination with increased levels of pro-inflammatory cytokines. In the context of a disturbance of the monocyte compartment, we report an impaired switch from inflammatory M1 to regenerative M2 macrophages in the lungs. Furthermore, an impaired signal transduction of the splenic response to the inflammatory reflex can be reported in obese mice. These mice exhibited fewer ChAT-expressing CD4^+^ T cells as well as a limited response of macrophages to nicotine treatment.

Obesity is a burden for the immune system leading to changes of immune cells due to chronic inflammation (57–59), which was also described for the DIO mouse model used in our study (60, 61). During the response to a combined thoracic and muscle trauma, DIO leads to the accumulation of pro-inflammatory immune cell populations at defined time points after trauma and a prolongation of a pro-inflammatory phase in general. These differences can be detected in the blood, the spleen, as well as in the traumatized tissue. Neutrophils, the first in line to respond to injury in the blood (62, 63), showed increased levels of neutrophils during initial time points post trauma, whereas obese mice exhibited prolonged accumulation of neutrophils compared to lean mice, which returned to the baseline level earlier after trauma. This prolonged and increased presence of neutrophils in obese mice may explain increased cytokine levels 6 h post trauma, since pro-inflammatory (IL-1α, IL-17A, and IL-6) or immunomodulatory (IFN-γ, IL-10, and IL-12) cytokines are released by neutrophils during activation in inflammatory settings (64). While neutrophils infiltrating the lung did not indicate any differences between lean and obese mice, neutrophils infiltrating the muscle displayed an increase as well as prolonged presence of neutrophils in the traumatized tissue. The described observation regarding increased levels of pro-inflammatory cytokines during the trauma response in a DIO mouse model contrasts with observations that were made in obese individuals after a severe trauma (65) who displayed decreased levels compared to normal weight individuals.

Besides neutrophils, monocytes presented a bimodal response in lean mice, involving an early increase of monocytes in the peripheral blood 1 h and a secondary increase 72 h post trauma. This elevated number of circulating monocytes is accompanied by reduced numbers of anti-inflammatory Ly6C^lo^ monocytes in the spleen. Strikingly, this secondary response is not detectable in obese mice, suggesting that the migration of Ly6C^lo^ monocytes from the spleen to the periphery is hindered in obese mice. Generally, the spleen is an important reservoir for monocytes (26, 66) as has also been shown in a mouse model for muscle dystrophy (27). In the context of monocyte migration and increased levels of circulating Ly6C^hi^ monocytes, high-mobility group box 1 (HMGB1) is an important contributor to inflammation. It has been shown that HMGB1 is an important player in inflammatory pathways (67) and the elucidation of the influence of HMGB1 in obesity-related inflammation would be intriguing for further studies.

From the onset, obese mice exhibited high levels of blood monocytes, which is in accordance with observations of monocytosis in obesity caused by IL-1β production of the adipose tissue (68). This increased level of monocytes did not change during early time points and an additional accumulation of pro-inflammatory Ly6C^hi^ monocytes was detected in the peripheral blood, traumatized muscle, and lung of obese mice 6 h post trauma. In addition, these monocytes express both CCR2 and CD62L. MCP-1 binds to CCR2 and is responsible for the migration of monocytes and macrophages to the site of inflammation by following the CCL2 gradient (69, 70). CD62L has been shown to be important for rolling efficiency and has a key role in regulating recruitment of monocytes to lymphoid tissues (71, 72) and can be shed during early activation (73, 74). The importance of CD62L in different implications of obesity, namely, nonalcoholic steatohepatitis (NASH) has been recently shown (75) in mice and humans.

CCR2^+^CD62L^+^ monocytes are also detectable in the tissue of obese mice 6 h and in lean mice during the secondary response 72 h post trauma. On the contrary, the increase of monocytes in the traumatized tissue of lean mice is independent from CD62L since both CCR2^+^CD62L^+^ and CCR2^+^CD62L^-^ monocytes accumulated in the lung of lean mice. These findings are similar to macrophages from obese and lean mice at 6 h and 72 h post trauma, respectively, which leads to the hypothesis that these macrophages are derived from blood monocytes. The prolonged presence of pro-inflammatory monocytes in the peripheral blood and the absence of a secondary response in obese mice contribute to a delayed switch from M1 to M2 macrophages. Lean mice entered the regeneration phase earlier, defined by phenotypically regenerative macrophages, which were not detectable in the lung tissue of obese mice (192 h).

The inflammatory reflex embodies a neural reflex circuit that can regulate inflammation processes to prevent damaging properties occurring from inflammation (52, 53). In several human studies, obesity has been linked to decreased vagus nerve activity (76–78) and the implication of the inflammatory reflex in linking immunity and metabolism was substantially reviewed by Pavlov and Tracey (53). Our study did not directly investigate the activity of the vagus nerve; however, two of the most important components, ChAT-expressing CD4^+^ T cells and signaling *via* the α7nAChR of macrophages, were examined. A previous study demonstrated that ChAT-expressing CD4^+^ T cells are crucial for the neuronal circuit (54), since the nerve fibers of the vagus nerve in the spleen lack the cholinergic machinery needed for acetylcholine production. Here, we demonstrated that the percentage of ChAT-expressing CD4^+^ T cells in the spleen of obese mice were reduced, indicating a potential impairment of signal transduction after vagus nerve stimulation. In addition, the treatment of LPS-stimulated splenocytes with various concentrations of nicotine led to decreased TNF-α secretion by macrophages from lean mice. This was not detected in splenocytes from obese mice. In this context, nicotine was used to mimic the α7nAChR signaling in macrophages comparable to vagus nerve stimulation (55, 56, 79). To our knowledge we are the first to report a reduced presence of ChAT-expressing CD4^+^ T cells and a reduced response to stimulation of the α7nAChR of macrophages in obese mice, which indicates an impaired response to the inflammatory reflex, which is supposed to diminish inflammatory processes. This could explain the extended inflammatory phase during the immune response of obese mice.

We acknowledge that this study has several limitations. First, the data and experiments presented as part of this study include *ex vivo* experiments although dealing with biologic phenomena that may be used for therapeutic intervention of obese trauma patients. Secondly, the study observation time was set to 192 h, which cannot exclude the idea that obese mice might generate regenerative macrophages at later time points. Finally, in the present mouse model, a sterile trauma induction was performed, in contrast to humans enduring a thoracic trauma as part of a polytrauma, most likely, acquire an unsterile trauma. Nevertheless, it will be interesting to transfer these findings to the human, especially with a focus on the impairment of the inflammatory reflex. These are important outcomes for future investigations.

In conclusion, we provide novel insights into how obesity influences an immune response after combined lung and muscle trauma. In this context, obese mice show an impaired signal transduction of the inflammatory reflex in the spleen without the additional trigger of a trauma. Furthermore, we were able to show differences in the immune response after trauma between lean and obese mice with a focus on the innate immune response. The described differences include prolonged circulation of neutrophils as well as inflammation-associated CCR2^+^CD62L^+^Ly6C^hi^ monocytes. Disturbances in the monocytic compartment provoke changes in the migration behavior of monocytes to the traumatized tissue and result in an impaired switch from inflammatory M1 to regenerative M2 macrophages in the traumatized lung of obese mice. However, further experimental investigations are needed to address how obesity influences the immune system in humans with trauma to provide a solid basis for the development of novel treatments.

## Data Availability Statement

The raw data supporting the conclusions of this article will be made available by the authors, without undue reservation.

## Ethics Statement

The animal study was reviewed and approved by Regierungspräsidium Tübingen Ulm University/license numbers: 1183 and 1493.

## Author Contributions

Study design by FG, MW, TB, LE, JB, and UK. Supervision by MW, TB, LE, and UK. PX established the mouse model. FG, AG, and PX performed the animal experiments. FG, AG, and AR performed the cytometry stainings, stimulations, and Legendplex assays. FG performed the data analysis. FG, TB, and UK wrote the paper. AG, AR, JB, and MW substantially revised the manuscript. All authors contributed to the article and approved the submitted version.

## Funding

MW and UK were supported by the Deutsche Forschungsgemeinschaft (DFG) as part of the SFB1149 “Danger Response, Disturbance Factors and Regenerative Potential after Acute Trauma” (251293561, project B04). FG, AG, and AR participated in the International PhD Programme of the International Graduate School in Molecular Medicine Ulm (GSC270). TB was funded by the Nazarbayev University Faculty-Development Competitive Research Grants Program, reference: 280720FD1907.

## Conflict of Interest

The authors declare that the research was conducted in the absence of any commercial or financial relationships that could be construed as a potential conflict of interest.

## Publisher’s Note

All claims expressed in this article are solely those of the authors and do not necessarily represent those of their affiliated organizations, or those of the publisher, the editors and the reviewers. Any product that may be evaluated in this article, or claim that may be made by its manufacturer, is not guaranteed or endorsed by the publisher.
