# Supplementary material for: Obesity Prolongs the Inflammatory Response in Mice After Severe Trauma and Attenuates the Splenic Response to the Inflammatory Reflex
Source: Front Immunol. 2021 Nov 15;12:745132. doi: 10.3389/fimmu.2021.745132 (PMC8634681; doi:10.3389/fimmu.2021.745132)
Supplement: Supplementary file 1 [file DataSheet_1.pdf]

Supplementary Material

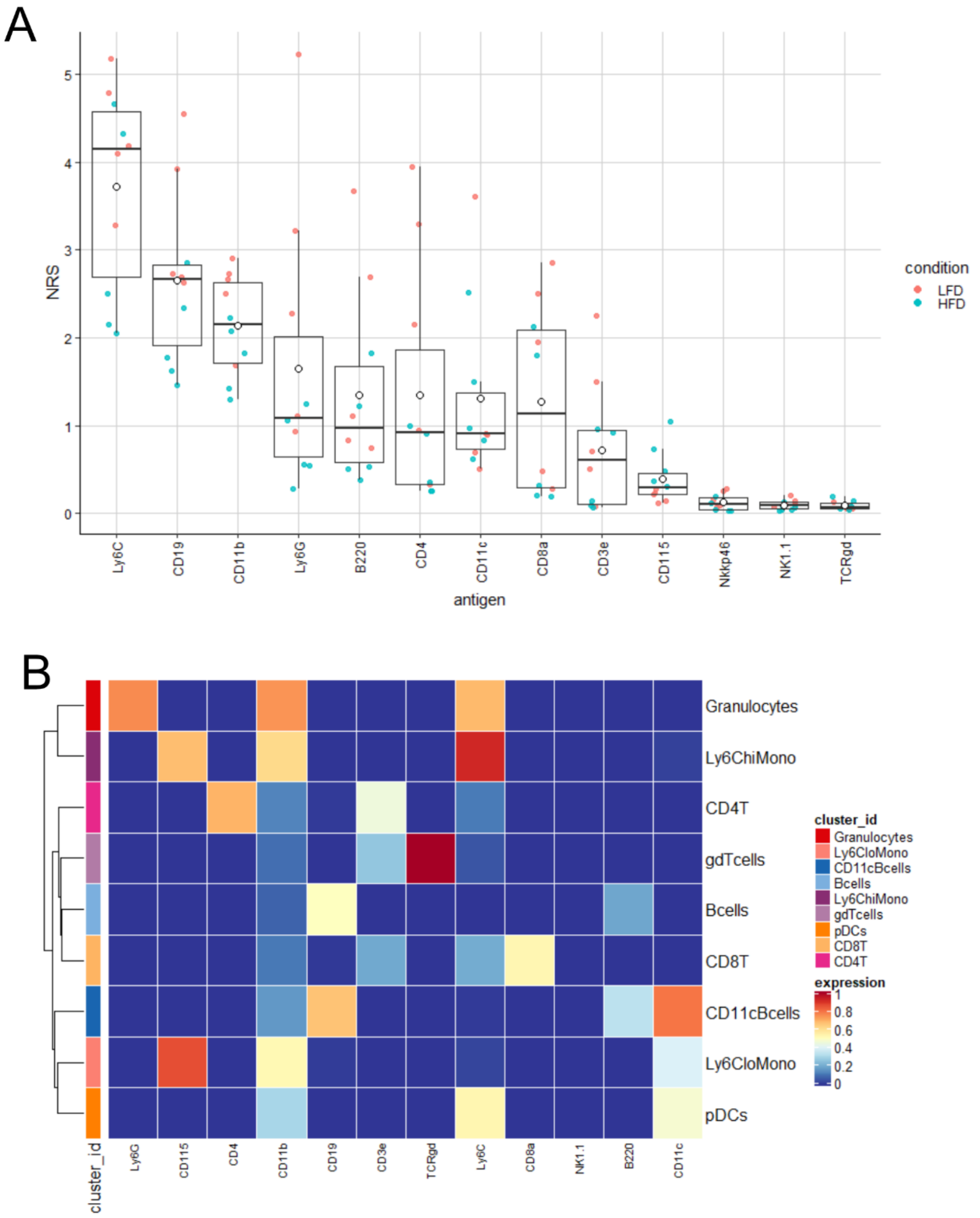

Suppl. figure 1: Additional information regarding unsupervised baseline comparison to evaluate the influence of diet induced obesity (DIO) on the immune system. (A) Markers used for unsupervised clustering by a self-organizing map (FlowSOM) ranked by highest non redundancy

scores (NRS), separated by the received diet into lean (LFD) and obese (HFD) mice. (B) Expression of markers used for clustering for the respective immune cell cluster.

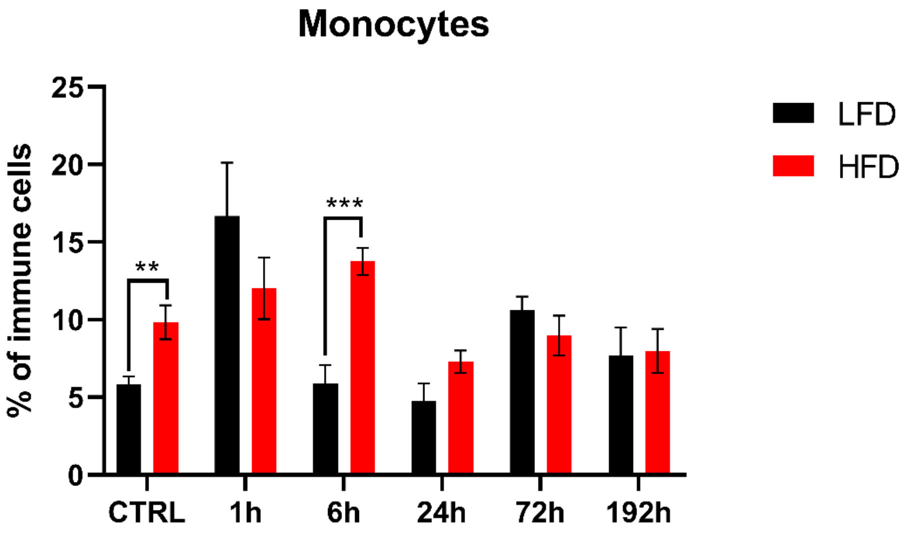

Suppl. figure 2: Percentages of circulating monocytes regarding all immune cells in lean (LFD) and obese (HFD) mice analyzed by flow cytometry. % of immune cells refers to the gate identifying the cells after data clean-up as described in Suppl. figure 6. Statistics: two-way ANOVA with an uncorrected Fisher‘s LSD test as follow-up was used to compare the level to the respective CTRL, significance indicators are displayed directly above the bar; unpaired two-tailed Student‘s t test was used for comparison of lean and obese mice at a specific timepoint, significance indicators are displayed above a connector line. ** indicates $p \leq 0.01$, *** indicates $p \leq 0.001$. Sample sizes: LFD CTRL = 8, LFD 1 h = 7, LFD 6 h = 5, LFD 24 h = 5, LFD 72 h = 5, LFD 192 h = 8, HFD CTRL = 7, HFD 1 h = 5, HFD 6 h = 5, HFD 24 h = 6, HFD 72 h = 5, HFD 192 h = 5. Data is displayed as mean ± SEM.

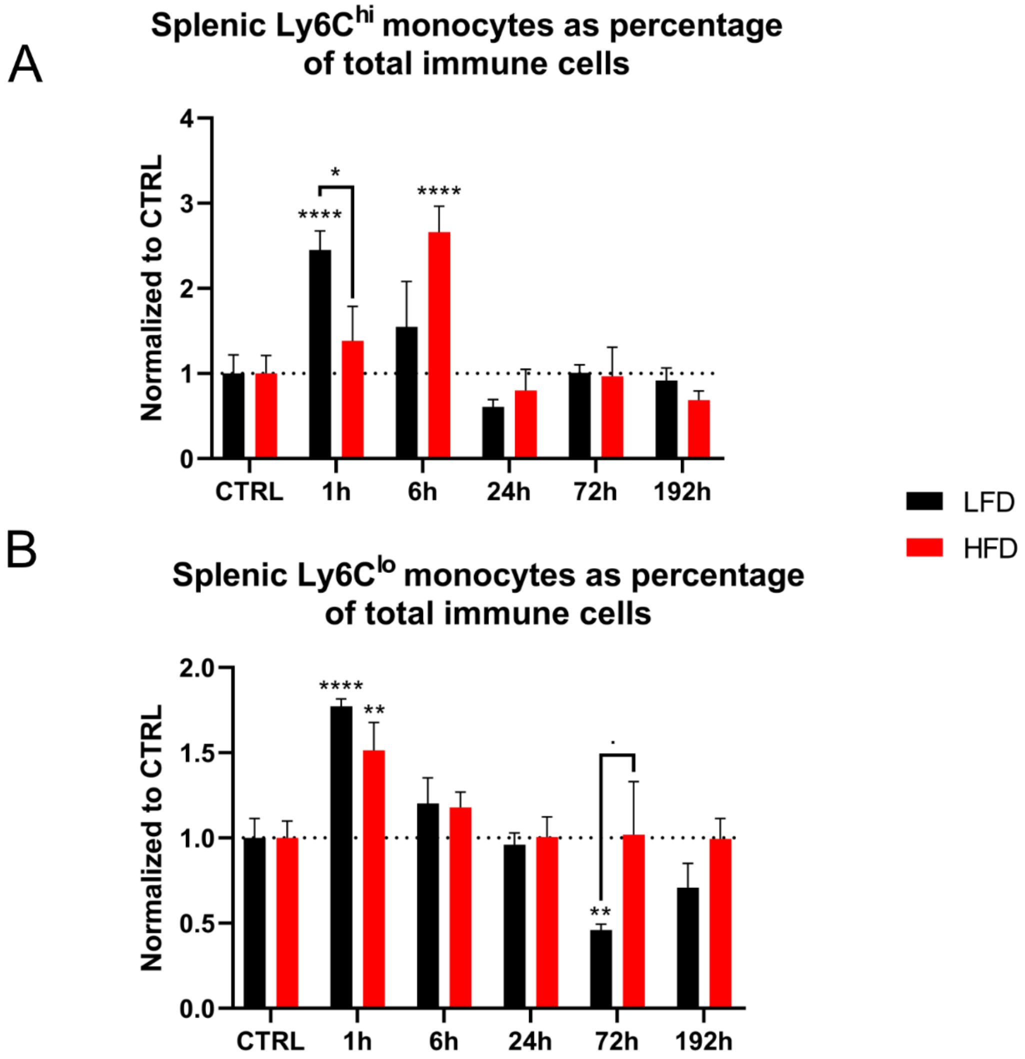

Suppl. figure 3: Myeloid cells in the spleen during the first 192 h analysed by flow cytometry. Baseline-corrected (ratio) timelapse for Ly6C^{hi} monocytes (A) and Ly6C^{lo} monocytes (B). Statistics: two-way ANOVA with an uncorrected Fisher's LSD test as follow-up was used to compare the level to the respective CTRL, significance indicators are displayed directly above the bar; unpaired two-tailed Student's t test was used for comparison of lean and obese mice at a specific timepoint, significance indicators are displayed above a connector line.· indicates $p \leq 0.1$, * indicates $p \leq 0.05$, ** indicates $p \leq 0.01$, **** indicates $p \leq 0.0001$. Sample sizes: LFD CTRL = 13, LFD 1 h = 6, LFD 6 h = 5, LFD 24 h = 5, LFD 72 h = 6, LFD 192 h = 6, HFD CTRL = 14, HFD 1 h = 5, HFD 6 h = 5, HFD 24 h = 7, HFD 72 h = 5, HFD 192 h = 8. Data is displayed as mean ± SEM.

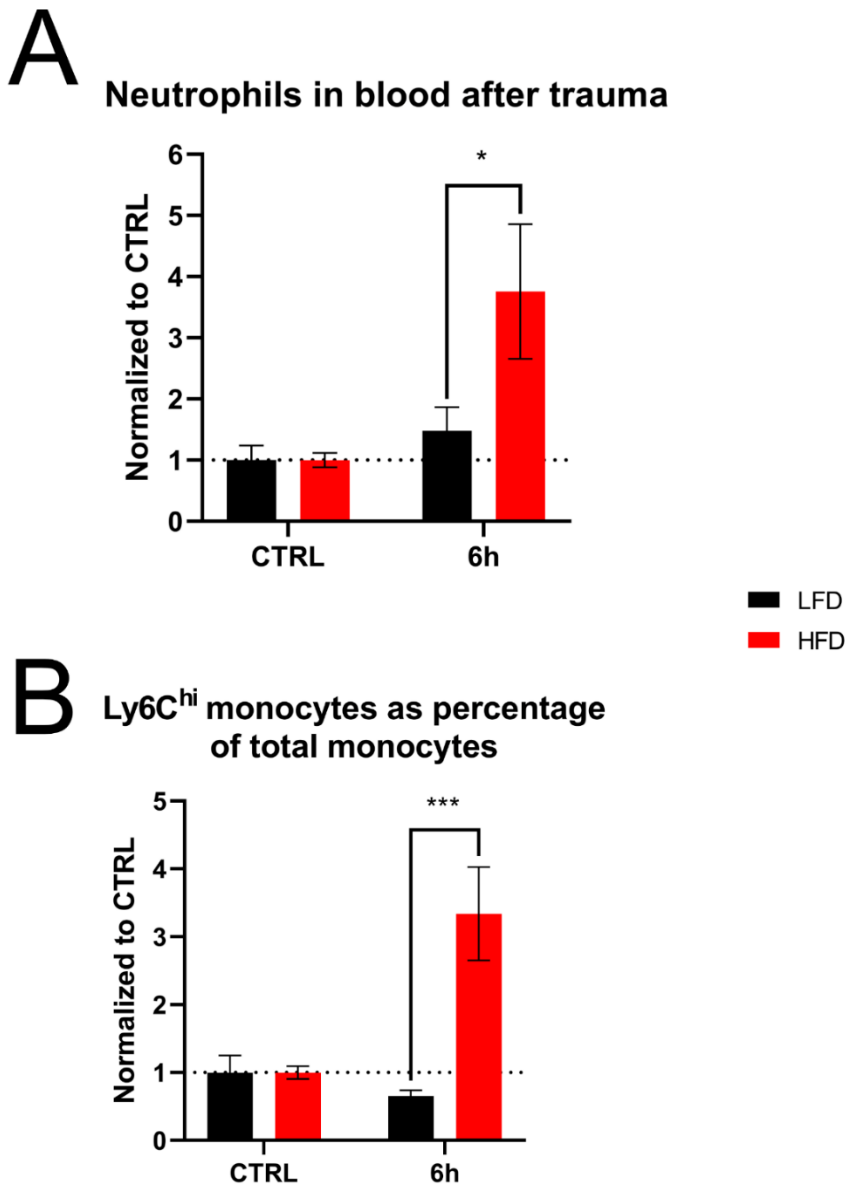

Suppl. figure 4: Mass cytometry data to confirm the results achieved by flow cytometry regarding increased levels of circulating neutrophils and pro-inflammatory monocytes 6 h post trauma in the blood of obese mice. Baseline-corrected (ratio) timelapse for neutrophils (A), and Ly6C^{hi} monocytes as percentages of total CD115^{+} monocytes. Statistics: two-way ANOVA with an uncorrected Fisher‘s LSD test as follow-up was used to compare the level to the respective CTRL, significance indicators are displayed directly above the bar; unpaired two-tailed Student‘s t test was used for comparison of lean and obese mice at a specific timepoint, significance indicators are displayed above a connector line. * indicates $p \leq 0.05$, *** indicates $p \leq 0.001$. Sample sizes: LFD CTRL = 5, LFD 6 h = 6, HFD CTRL = 5, HFD 6 h = 4. Data is displayed as mean ± SEM.

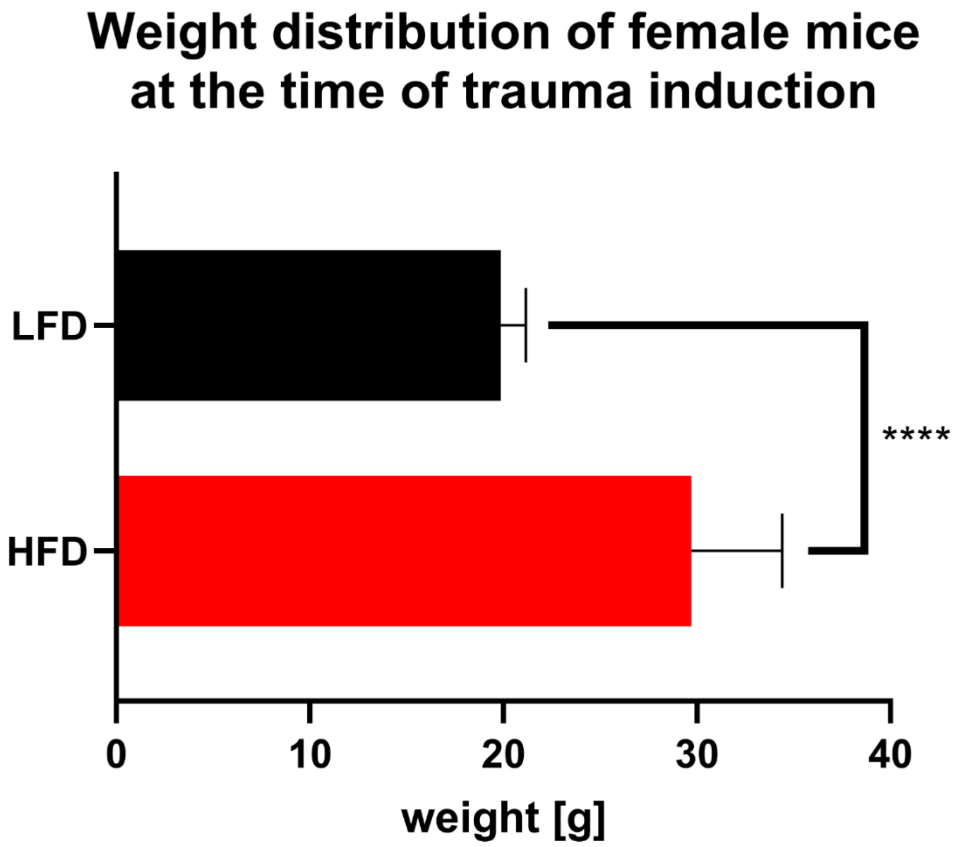

Suppl. figure 5: Weight distribution of female C57BL/6J mice at the time of trauma induction for lean (LFD) and obese (HFD) mice. Statistics: unpaired two-tailed Student‘s t test was used for comparison of lean and obese mice. **** indicates $p \leq 0.0001$. Sample sizes: LFD = 91, HFD = 74. Data is displayed as mean ± SD.

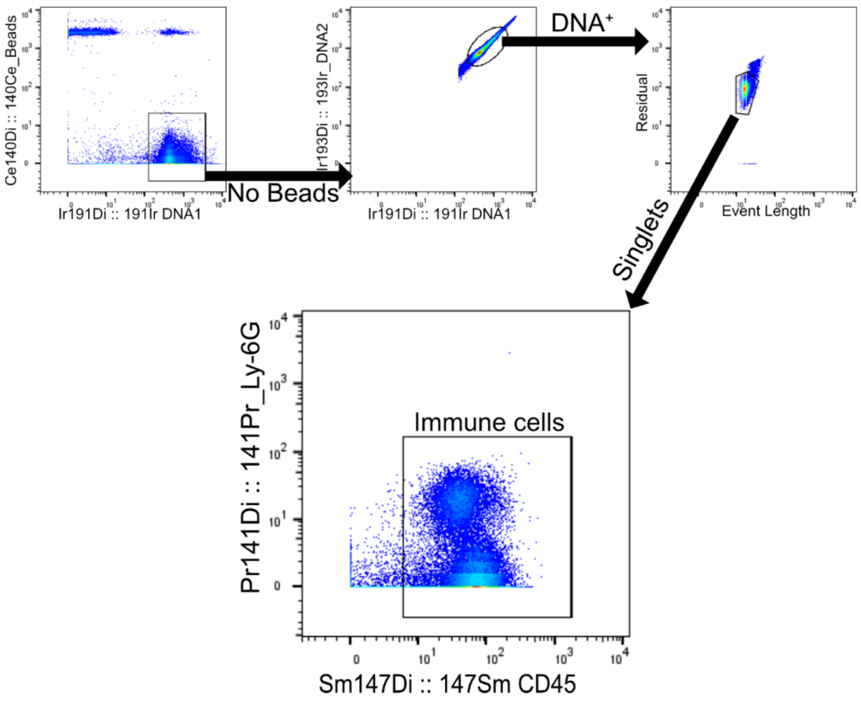

Suppl. figure 6: Clean-up gating scheme for mass cytometry data. Beads are actively gated out of the analysis using DNA1 staining, followed by the identification of DNA double-positive Events (191Ir DNA1 and 193Ir DNA2). Singlets are identified by Event length and residual utilizing gaussian gating. CD45$^+$ cells were identified as immune cells and are the basis for all analyses shown in this study.

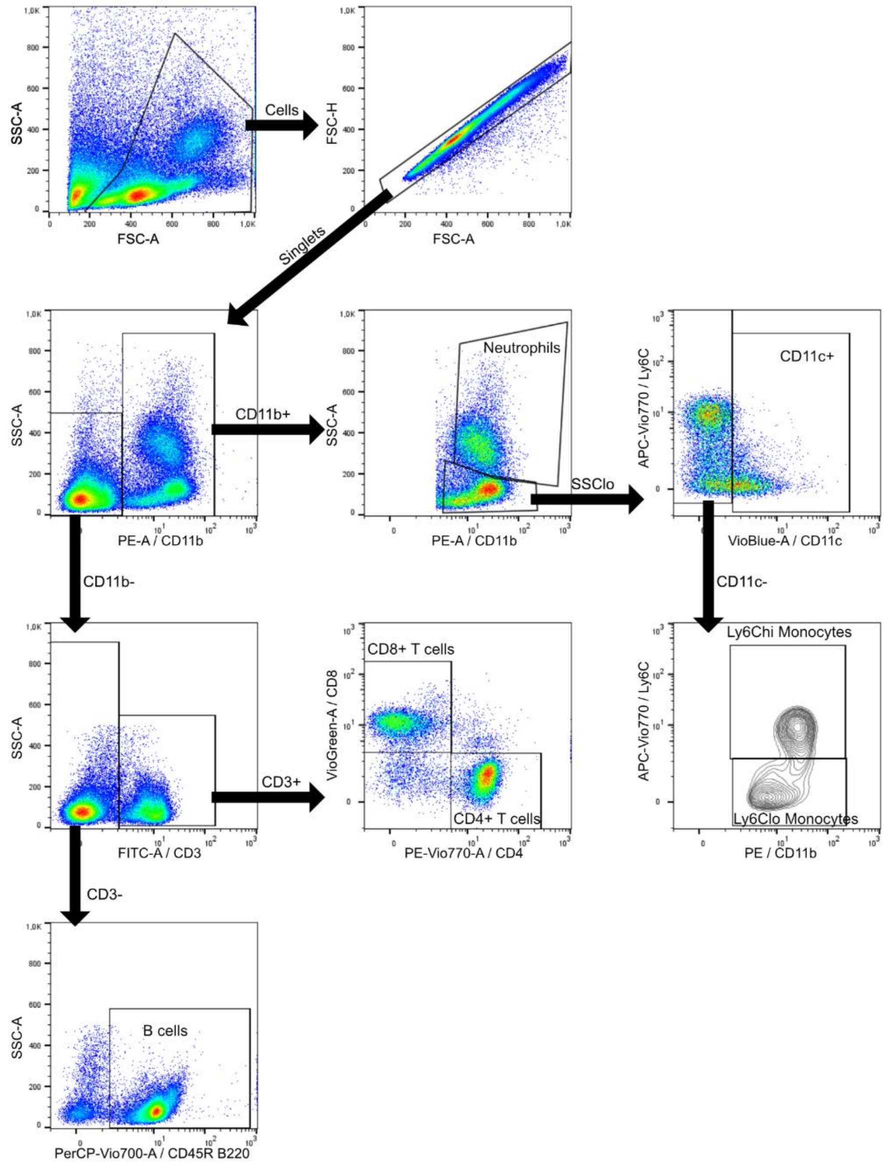

Suppl. figure 7: Gating scheme for flow cytometry data used for phenotyping of immune cell populations during the trauma response. This gating scheme was used for blood and spleen samples that were harvested during defined time points (CTRL, 1 h, 6 h, 24 h, 72 h, 192 h) of the trauma response.

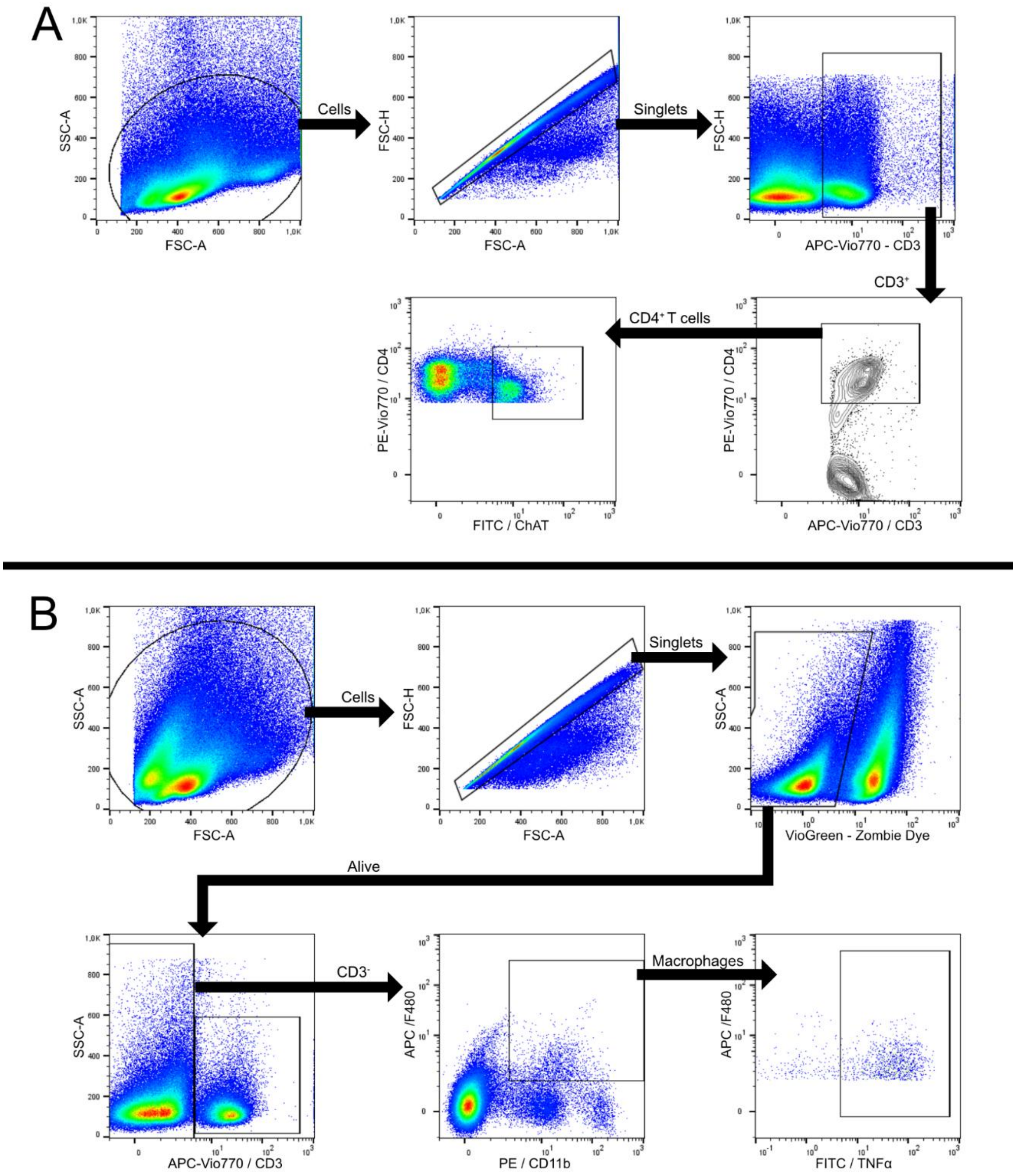

Suppl. figure 8: Gating scheme for flow cytometry data used for phenotyping of immune cell populations in the spleen at baseline with the influence of the diet. (A) depicts the gating scheme utilized to identify ChAT expressing $CD4^+$ T cells. (B) shows the gating scheme used to measure produced TNFα after LPS stimulation of splenocytes.

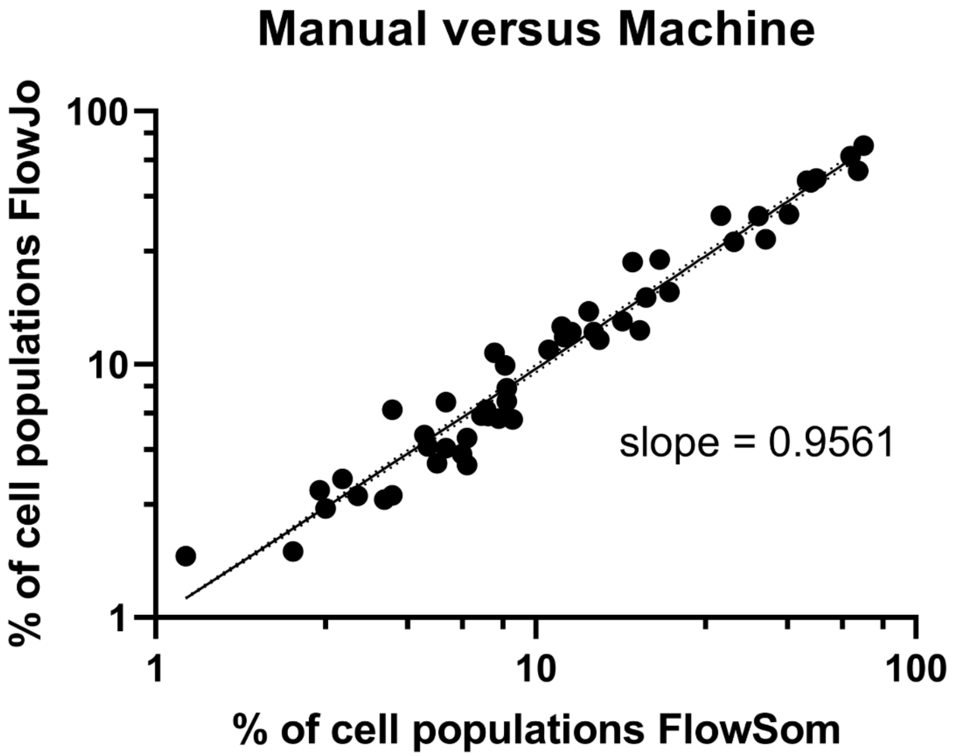

Suppl. figure 9: Comparison of percentages of immune cell populations identified by manual gating using FlowJo and unsupervised clustering utilizing a self-organizing map (FlowSOM). Comparison was performed using CTRL mice samples from the analysis depicted in figure 1. Statistics: Simple linear regression, while forcing the curve to go through x = 1 and y = 1.

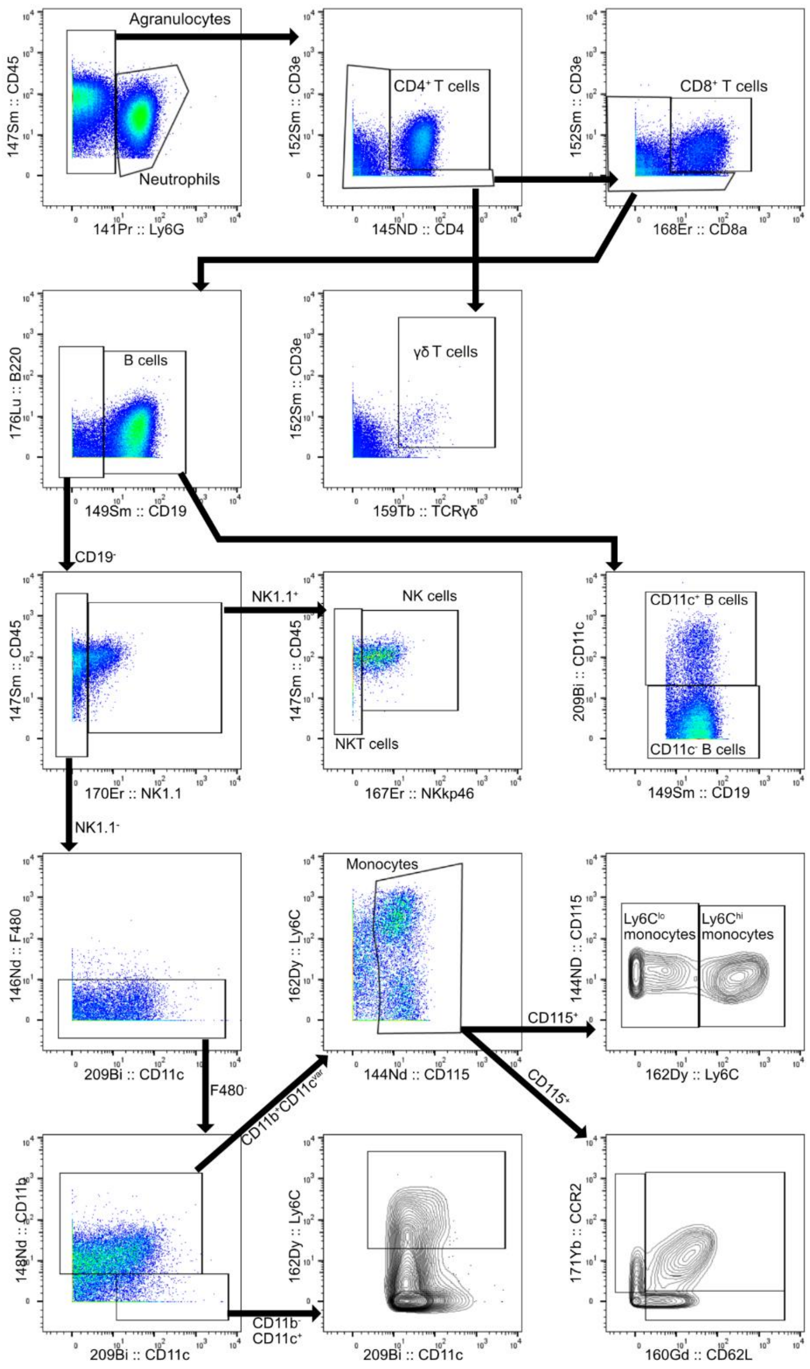

Suppl. figure 10: Gating scheme for mass cytometry data of blood samples. Gating scheme starts after clean-up of FCS files, which is depicted in suppl. Fig 6.

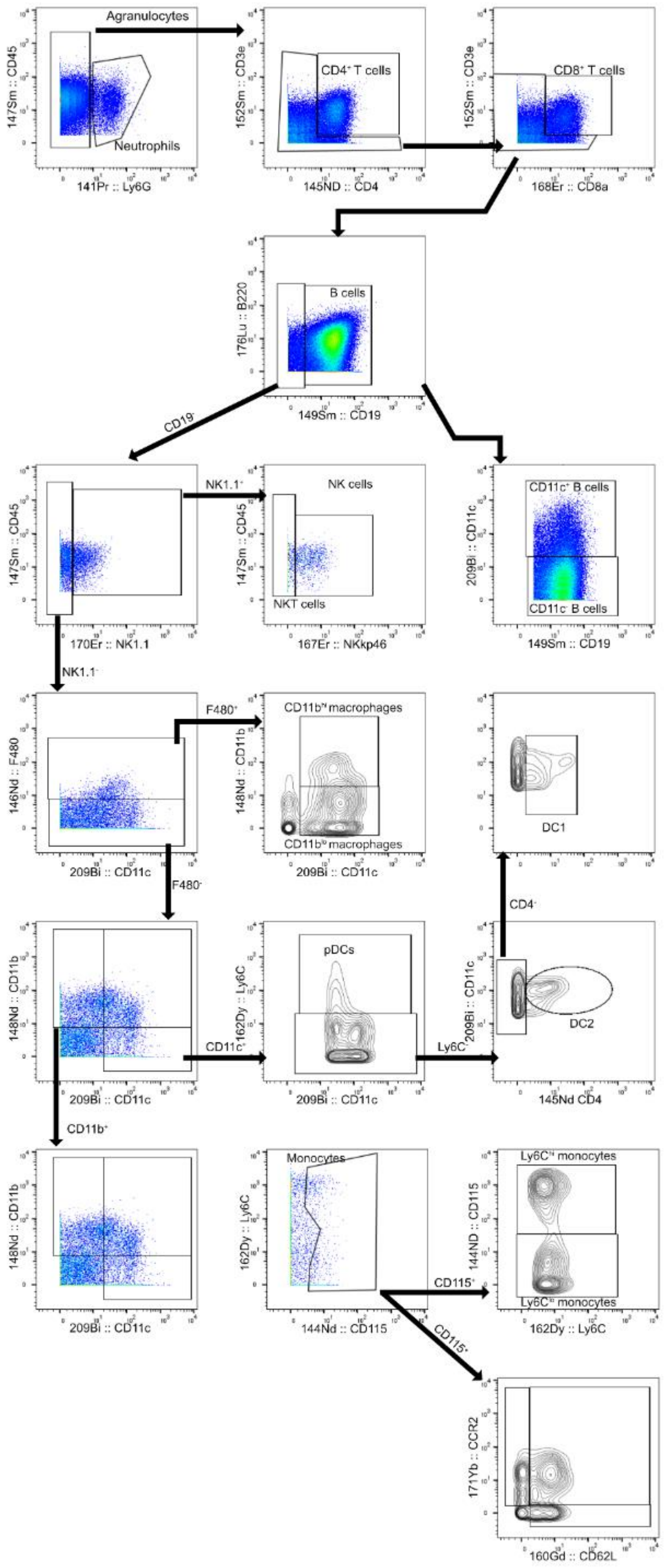

Suppl. figure 11: Gating scheme for mass cytometry data of spleen samples. Gating scheme starts after clean-up of FCS files, which is depicted in suppl. Fig 6.

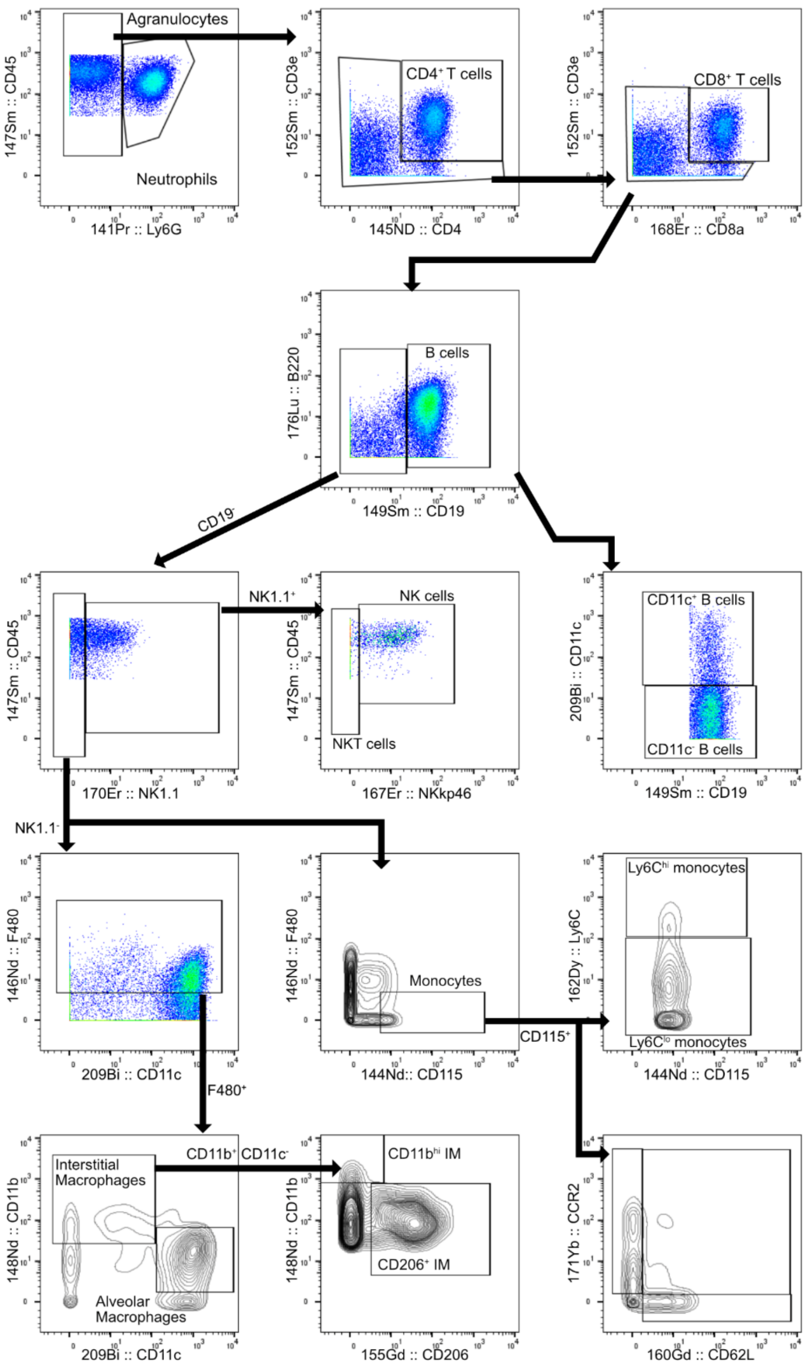

Suppl. figure 12: Gating scheme for mass cytometry data of lung samples. Gating scheme starts after clean-up of FCS files, which is depicted in suppl. Fig 6.

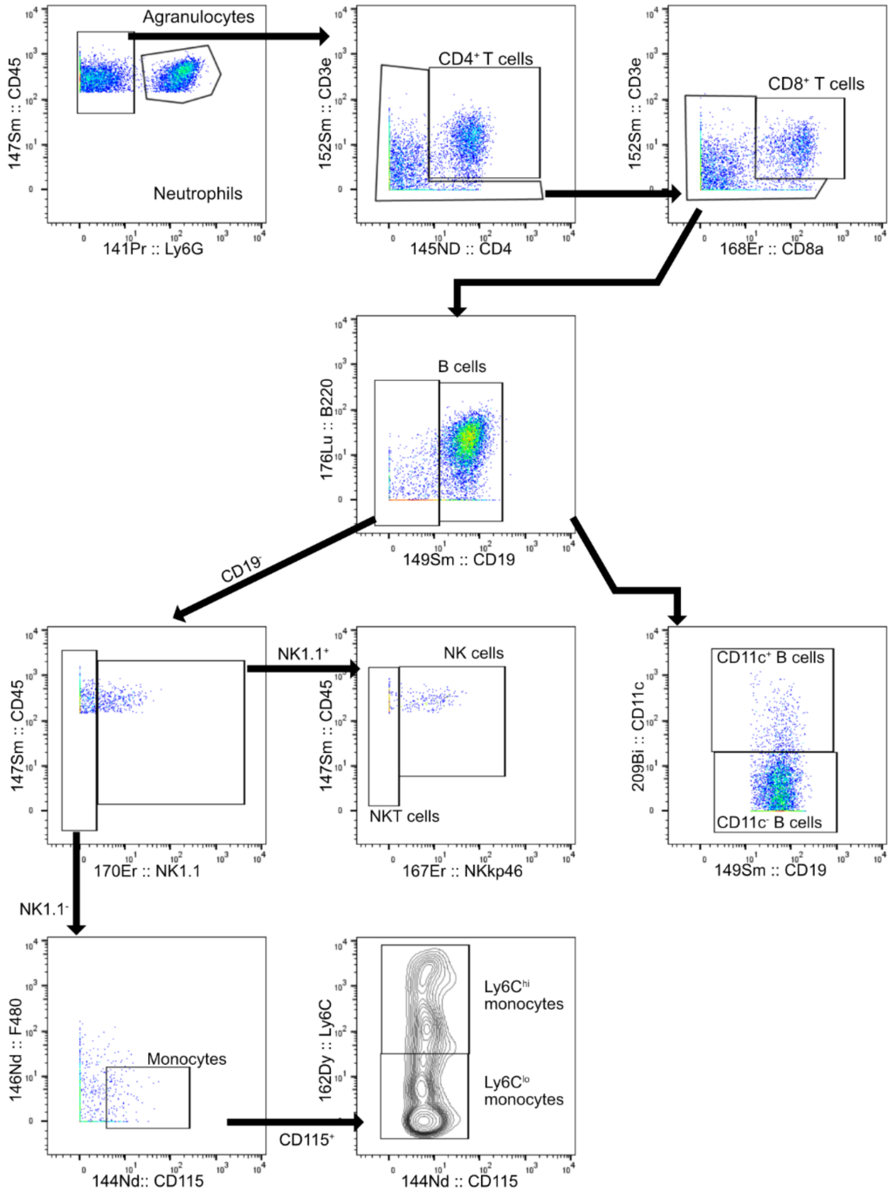

Suppl. figure 13: Gating scheme for mass cytometry data of muscle samples. Gating scheme starts after clean-up of FCS files, which is depicted in suppl. Fig 6.

Suppl. R Code 1: R code used for the analysis of CyTOF data. R code and construct is based on the CyTOF workflow procedure as described by Nowicka et al. (Nowicka et al. 2017).

```
RNGversion("3.5.3")

## Set Workingdirection

setwd("XXX")

##General Information

md <- read_excel("XXX")

head(data.frame(md))


##FlowDataset

fs = read.flowSet(md$file_name, transformation = FALSE, truncate_max_range = FALSE)

##Panel

panel <- read_excel("XXX")

head(data.frame(panel))

# spot check that all panel columns are in the flowSet object

all(panel$fcs_colname %in% colnames(fs))

# specify levels for conditions & sample IDs to assure desired ordering

md$condition <- factor(md$condition, levels = c("LFD", "HFD"))

md$sample_id <- factor(md$sample_id, levels = md$sample_id[order(md$condition)])

# construct SingleCellExperiment

sce <- prepData(fs, panel, md, features = panel$fcs_colname)

#Generate Plots based on expression

p <- plotExprs(sce, color_by = "condition")

p$facet$params$ncol <- 4

p
```

```
#Number of cells per file

n_cells(sce) # or, equivalently, `metadata(sce)$experiment_info$n_cells`

plotCounts(sce, color_by = "condition")

#MDS plot

CATALYST::plotMDS(sce, color_by = "condition")

plotExprHeatmap(sce, bin_anno = TRUE, row_anno = TRUE)

#Markers based on non-redundancy score

plotNRS(sce, features = type_markers(sce), color_by = "condition")

#FlowSOM

set.seed(1234)

sce <- cluster(sce, features = type_markers(sce), xdim = 10, ydim = 10, maxK = 50, seed = 1234)

#Marker Heatmap

plotClusterHeatmap(sce, hm2 = NULL, k = "meta50", m = NULL, cluster_anno = TRUE,
draw_freqs = TRUE)

plotClusterExprs(sce, k = "meta50", features = "type")

plotClusterHeatmap(sce, hm2 = "Ly6C", k = "meta50", draw_freqs = TRUE)

# run t-SNE/UMAP

set.seed(1234)

sce <- runDR(sce, dr = "TSNE", cells = 1000, features = "type")

sce <- runDR(sce, dr = "UMAP", cells = 1e5, features = "type")

plotDR(sce, "TSNE", color_by = "meta50")

plotDR(sce, "UMAP", color_by = "meta50")

p1 <- plotDR(sce, "TSNE", color_by = "meta50") + theme(legend.position = "none")

p2 <- plotDR(sce, "UMAP", color_by = "meta50")

lgd <- get_legend(p2 guides(color =  guide_legend(ncol = 2, override.aes = list(size = 3))))

p2 <- p2 + theme(legend.position = "none")
```

```
plot_grid(p1, p2, lgd, nrow = 1, rel_widths = c(5, 5, 2))

## Facet per sample

plotDR(sce, "UMAP", color_by = "meta50")  + facet_wrap("condition") guides(color =
guide_legend(ncol = 2, override.aes = list(size = 3)))



## Facet per condition

plotDR(sce, "TSNE", color_by = "meta50") + facet_wrap("condition") + guides(color =
guide_legend(ncol = 2, override.aes = list(size = 3))

#Heatmap of median expression



## Rename clusters

merging_table1 <- read_excel("XXX.xlsx")

head(data.frame(merging_table1))

# convert to factor with merged clusters in desired order

merging_table1$new_cluster <- factor(merging_table1$new_cluster, levels =
c("Ly6CloF480","Ly6ChiF480","Ly6Clo","Ly6Chi","Ly6Cint","CD11c+Ly6C+Macro","Ly6ChiMa
cro","Ly6CloMacro","CD11c+Macro","Ly6CNK","NK","Macrophages","CD11bloMacro","CD11bh
iMacro","DC1","DC2","Granulocytes", "Double positives","Ly6CloMono","CD11c+CD11b-
","CD11cBcells","Bcells","Ly6ChiMono","CD11b","gdTcells","Ly6Cpos","pDCs","CD8T","gdT","
CD4T","IM","AM"))

# apply manual merging

sce <- mergeClusters(sce, k = "meta50”, table = merging_table1, id = "merging1")

plotCodes(sce, k = "merging1")

plotDR(sce, "UMAP", color_by = "CCR2")

plotDR(sce, "TSNE", color_by = "merging1")

p1 <- plotDR(sce, "TSNE", color_by = "merging1") + theme(legend.position = "none”)

p2 <- plotDR(sce, "UMAP", color_by = "merging1")

lgd <- get_legend(p2 + guides(color =  guide_legend(ncol = 2, override.aes = list(size = 3))))

p2 <- p2 + theme(legend.position = "none")
```

```
plot_grid(p1, p2, lgd, nrow = 1, rel_widths = c(5, 5, 2))

plotDR(sce, "UMAP", color_by = "merging1") + facet_wrap("condition") + guides(color =
guide_legend(ncol = 2, override.aes = list(size = 3)))

plotDR(sce, "TSNE", color_by = "merging1") + facet_wrap("condition") + guides(color =
guide_legend(ncol = 2, override.aes = list(size = 3)))

plotClusterHeatmap(sce, k = "merging1")
```
